# A comparative analysis of in vitro toxicity of diesel exhaust particles from combustion of 1st- and 2nd-generation biodiesel fuels in relation to their physicochemical properties—the FuelHealth project

**DOI:** 10.1007/s11356-017-9561-9

**Published:** 2017-07-03

**Authors:** Anna Lankoff, Kamil Brzoska, Joanna Czarnocka, Magdalena Kowalska, Halina Lisowska, Remigiusz Mruk, Johan Øvrevik, Aneta Wegierek-Ciuk, Mariusz Zuberek, Marcin Kruszewski

**Affiliations:** 1 Department of Radiobiology and Immunology, Institute of Biology, Jan Kochanowski University, 15 Swietokrzyska Str., 25-406 Kielce, Poland; 2 Center for Radiobiology and Biological Dosimetry, Institute of Nuclear Chemistry and Technology, 16 Dorodna Str., 03-195 Warsaw, Poland; 3 Automotive Industry Institute, 55 Jagiellońska Str., 03-301 Warsaw, Poland; 4 Faculty of Production Engineering, Warsaw University of Life Sciences, 166 Nowoursynowska Str., 02-787 Warsaw, Poland; 5 Division of Environmental Medicine Norwegian Institute of Public Health, Norwegian Institute of Public Health, P.O. Box 4404 Nydalen, 0403 Oslo, Norway; 6 Department of Molecular Biophysics, Faculty of Biology and Environmental Protection, University of Łódź, 141/143 Pomorska St., 90-236 Lodz, Poland; 7 Independent Laboratory of Molecular Biology, Institute of Rural Health, Jaczewskiego 2, 20-090 Lublin, Poland

**Keywords:** Diesel exhaust particles, Physicochemical characterization, 1st- and 2nd-generation biodiesel fuels, Cellular binding/uptake, Cytotoxicity, Reactive oxygen species, Gene expression

## Abstract

Biodiesels represent more carbon-neutral fuels and are introduced at an increasing extent to reduce emission of greenhouse gases. However, the potential impact of different types and blend concentrations of biodiesel on the toxicity of diesel engine emissions are still relatively scarce and to some extent contradictory. The objective of the present work was to compare the toxicity of diesel exhaust particles (DEP) from combustion of two 1st-generation fuels: 7% fatty acid methyl esters (FAME; B7) and 20% FAME (B20) and a 2nd-generation 20% FAME/HVO (synthetic hydrocarbon biofuel (SHB)) fuel. Our findings indicate that particulate emissions of each type of biodiesel fuel induce cytotoxic effects in BEAS-2B and A549 cells, manifested as cell death (apoptosis or necrosis), decreased protein concentrations, intracellular ROS production, as well as increased expression of antioxidant genes and genes coding for DNA damage-response proteins. The different biodiesel blend percentages and biodiesel feedstocks led to marked differences in chemical composition of the emitted DEP. The different DEPs also displayed statistically significant differences in cytotoxicity in A549 and BEAS-2B cells, but the magnitude of these variations was limited. Overall, it seems that increasing biodiesel blend concentrations from the current 7 to 20% FAME, or substituting 1st-generation FAME biodiesel with 2nd-generation HVO biodiesel (at least below 20% blends), affects the in vitro toxicity of the emitted DEP to some extent, but the biological significance of this may be moderate.

**Electronic supplementary material:**

The online version of this article (doi:10.1007/s11356-017-9561-9) contains supplementary material, which is available to authorized users.

## Introduction

Diesel exhaust emission (DEE) is considered a major source to urban air pollution and has been associated with exacerbation and/or development of respiratory problems, inflammation-related diseases, cardiovascular morbidity and mortality, as well as cancer occurrence in humans (Schwarze et al. 2013; Krewski et al. 2015; Manzetti and Andersen 2016). CO_2_ emissions from diesel engines also contribute to the enhancement of greenhouse effect, playing a major role in shaping the earth’s climate and global warming (Resitoglu et al. 2015). As an alternative to standard fossil diesel fuels, biodiesel offers a potentially attractive solution for reduction of greenhouse gases. Moreover, being made from renewable resources, it also addresses energy security concerns (Huang et al. 2012; US EPA 2011). Though neat biodiesel and biodiesel blends with fossil diesel fuels are currently used only in low amounts in Europe, vision plans have been proposed to increase the shear of biofuels considerably in the near future. According to the newest EU Directive 2015/1513, all EU countries must achieve at least 20% share of renewable energy in the overall energy consumption by 2020, including at least 10% share in transport fuels. Thus, the demand for biodiesel fuels continues to grow rapidly. However, the 1st-generation biodiesel fuels have received considerable criticism, as being responsible for rising food prices, having relatively low impact on greenhouse gas emissions, and direct and indirect impacts on land use change. This debate has pushed 2nd-generation biodiesel, using nonfood feedstocks, under the spotlight, with the hope that they will soon become fully commercialized and solve many issues concerning 1st-generation biodiesels (International Energy Agency Report 2008). The most common feedstock blends in EU are fatty acid methyl esters (FAME), rapeseed methyl ester (RME), soy methyl ester (SOE), and hydrotreated vegetable oils (HVO).

The choice of feedstock blend strongly influences the composition of biodiesel fuel, which in turn influences exhaust composition and potentially also human health effects from DEE exposure (Moser and Vaughn 2010; Steiner et al. 2013). By now, numerous laboratory studies have shown a consistent reduction for hydrocarbons (HC) and carbon mono-/dioxide (CO/CO_2_) emission with increasing concentration of biocomponent in fuel, although there was an increase in nitrogen oxides (NOx) (McCormick 2007). While biodiesel is expected to decrease polycyclic aromatic hydrocarbons (PAH) and nitro-polycyclic aromatic hydrocarbons (NPAH) emission due to the lower content of key PAH precursors (Karavalakis et al. 2010), information regarding other unregulated emissions with alternative fuels is limited and inconsistent. Ratcliff et al. (2010) reported that the use soybean-based biodiesel and biodiesel blends substantially reduced particle-associated PAH and NPAH emissions compared with pure diesel. However, Karavalakis et al. (2010) have shown that while PAH emissions were reduced, NPAH and oxy-PAH emissions increased with soy-based biodiesel. In contrast, Zou and Atkinson (2003) found that PAH emissions increased or were unchanged after combustion of canola oil-based biodiesel. Similarly, inconsistent emission patterns were observed for the content of aldehydes and ketones, showing that combustion of certain biodiesels resulted in significant increase, while others led to decreases of these pollutants (Fontaras et al. 2010). Of all regulated and nonregulated emissions of exhaust from biodiesel blends, only reduction in particulate matter (PM) was significant (Bünger et al. 2012). Nevertheless, the diminution of particulate mass with increasing blend percentage was often caused by the emission of more toxic smaller particles than larger particles (Surawski et al. 2011).

Knowledge on the physicochemical properties of diesel exhaust particles (DEP) has significant implication for biological response interpretation. Recent toxicological and epidemiological studies on the adverse health effects of biodiesel DEP showed inconsistent results (Mutlu et al. 2015). DEP from rapeseed and soy-based biodiesel fuel appear to have a stronger pro-inflammatory potential compared with DEP from conventional diesel fuels, both in vitro and in vivo (Swanson et al. 2009; Fukagawa et al. 2013; Gerlofs-Nijland et al. 2013). Cheung et al. (2009) reported that DEP from soy-based biodiesel elevated generation of reactive oxygen species (ROS) with increasing blend percentage. On the contrary, Hemmingsen et al. (2011) suggested that canola-based biodiesel particles were less toxic than conventional petrodiesel particles. The reported mutagenic effect of biodiesel particles as compared with regular diesel fuel has also been contradictory: lesser for rapeseed- and soy-based biodiesel-derived particles (Kado and Kuzmicky 2003) and higher for rapeseed-based biodiesel particles (Bünger et al. 2007). More recently, Jalava et al. (2010) reported the differences in the induction of DNA strand breaks between combustion particles from rapeseed biodiesel fuel and pure diesel. In summary, a comparison of the results of different toxicological studies for exhaust particles produced by biodiesel combustion is difficult because of differences in the used experimental approach, including age and type of diesel engine, drive cycle, feedstock blend, and its percentage in the blended fuel. Thus, when evaluating the biological response to exhaust particles, a proper and extensive physicochemical analysis of the particles should be an initial step. The objective of the present work was to compare the toxicity of different DEPs from combustion of 1st- and 2nd-generation biodiesel fuels in relation to their physicochemical properties. DEPs were produced by the 1.3 JTD engine (Euro V stage), fueled with three biodiesel fuels of commercial interest: the 1st-generation B7 biodiesel fuel (7% FAME), which is currently used in EU, the 1st-generation B20 biodiesel fuel (20% FAME), and the 2nd-generation SHB biodiesel fuel (7% FAME and 13% synthetic HVO). These biofuels were combusted under identical engine operation conditions, and emissions were evaluated during a certified test cycle. Detailed physicochemical characterizations of diesel exhaust particles were performed to investigate how the composition of three types of DEPs affects their biological effects in vitro, measured as cellular uptake kinetics, cell death response, total protein content, and the production of ROS in BEAS-2B and A549 cells. In addition, the expression of genes regulated during cellular responses to stress and xenobiotics was also evaluated to screen for possible molecular mechanisms of toxicity.

## Materials and methods

### Collection of DEPs

A Fiat Panda with a compression ignition engine 1.3 JTD (Common Rail 3rd-generation injection system; engine capacity, 1248 cm^3^; max power, 75 bhp; max torque, 190 Nm; production year, 2014), fulfilling the requirements of the Euro V stage was used as a DEP source. The engine was tested under controlled conditions on a chassis dynamometer (Schenck Komeg EMDY 48) at static engine speed and load, 1340 rpm and 45.7% respectively, corresponding to a constant vehicle speed of 43.75 km/h. Engine temperature were kept at 94 °C during the test cycle. The engine was fueled by three different mixtures of diesel oil and biocomponents: (1) 1st-generation biodiesel fuel “B7,” containing 7 vol.% FAME in diesel oil, (2) 1st-generation biodiesel fuel “B20,” containing 20 vol.% FAME in diesel oil, and (3) 2nd-generation biodiesel fuel “SHB,” containing 13 vol.% synthetic HVO (NExBTL, Neste Oil) and 7 vol.% FAME in diesel oil. The DEPs used in the present study were collected from the main diesel exhaust without diesel particle filter on PTFE-coated glass fiber filters (70 mm; Pallflex, Emfab filters, TX40HI20WW). The filters were weighted before and after sampling to determine the total particulate matter collected. For PAH content analysis, the filters were pooled and extracted with dichloromethane. Particles for in vitro experiments were scraped from the filters by using a clean stainless blade.

### Preparation of diesel exhaust particles (DEPs)

DEP-stock solutions were prepared by dispersion of 2 mg of particles in 1 mL of LHC-9 serum-free medium (for experiments with BEAS-2B cells) or in 1 mL of F12 Ham medium supplemented with 10% FCS (for experiments with A549 cells). DEP dispersions were then sonicated on ice using the Sonic Vibra Cell ultrasonic liquid processor (USA). Ultrasonic energy (3 kJ) was provided in pulses (30 s on, 10 s off) at 60% amplitude. Stock solutions were dispensed (100 μL) into sterile 1 mL cryogenic vials and stored at −20 °C. The samples were thawed before each set of experiments at 37 °C for 60 s, dispersed in the corresponding medium at a ratio of 1:10, and mixed prior to use (working solution).

### Nanoparticle tracking analysis (NTA)

Nanoparticle tracking analysis (NTA) was performed before each set of experiments with a NanoSight LM20 (NanoSight, Amesbury, UK), equipped with a sample chamber with a 640-nm laser. Working solutions of DEPs were diluted 1:4 in the corresponding medium and injected in the sample chamber. All measurements were performed at room temperature. The hydrodynamic size distribution of the DEP samples were analyzed using the NTA 2.0 Build 127 software.

### Zeta potential and polydispersity index measurements by DLS method

The zeta-potential of the DEP samples were measured at 25 °C in a folded capillary cell at 150 V and M3-PALS detection using noninvasive backscatter at 173° with an Avalanche photodiode, Q.E. > 50% at 633 nm (Malvern, Malvern Hills, UK). Working solutions were diluted 1:8 in the corresponding medium and measured in triplicate with 20 subruns. Zeta potentials were calculated using the Smoluchowski limit for the Henry equation with a setting calculated for practical use (*f* (ka) = 1.5). The polydispersity index (PDI) was obtained from the autocorrelation function. The default filter factor of 50% and the default lower threshold of 0.05 and upper threshold of 0.01 were used.

### Analysis of DEPs by transmission electron microscopy (TEM)

For each sample, a small drop of the DEP-working solution was placed onto the transmission electron microscopy (TEM) copper mesh coated with a thin polymeric support film. After evaporation of the solvent under vacuum, the size and shape of the particles were analyzed by transmission electron microscope JEOL 1200 EXII (JEOL, Japan) operating at an acceleration voltage of 120 kV. Digital images were recorded by CD camera SIS Morada 11 megapixels and processed using AnalySIS.

### Elemental analysis of DEPs by digital scanning electron microscopy (SEM) with energy dispersive X-ray spectroscopy (EDS)

The powdered DEP-samples were fixed to scanning electron microscopy (SEM) holder with the Quick Drying Silver Paint (Agar, UK) conductive glue and coated with thin layer of Au (about 10 nm) using a vacuum evaporator (JEE-4X, JEOL, Japan) to assure conductivity, protect the sample from heat destruction and to keep real parameters of the observed details. The samples were examined in a DSM 942 scanning electron microscope (Zeiss, Germany) in a secondary electron (SE) mode. Microscope parameters were set to high voltage (HV) = 10 kV and working distance (WD) = 6 mm. The elements present in investigated samples were determined using the energy dispersive X-ray spectrometry (EDS) using Quantax 400 (Bruker, Germany) system set to HV = 15 kV and WD = 20 mm.

### Separation and analysis of polycyclic aromatic hydrocarbons (PAHs) from particulate extracts

Separation and analysis of PAHs from particulate extracts was described in detail by Czarnocka and Odziemkowska (2016). Briefly, DEP samples were extracted into the mixture of acetone/hexane (1:4) in a horizontal shaker (10 min) and in an ultrasonic bath (2 × 25 min). The extracts (DEP-OE) were dehydrated using the anhydrous sodium sulfate (VI) solution, and PAHs were separated by solid phase extraction (SPE) on silica gel columns. The aliphatic hydrocarbons were eluted as the first fraction with hexane. Then, the cartridge was dried and the PAHs concentrated to a volume of 1 mL were eluted with dichloromethane (Super Purity Solvent). The solvent was changed to methanol (Super Purity Solvent) before injection. PAH content was measured by the Agilent 7890A GC System chromatograph coupled with a mass spectrometer MS 5975C using a low-polarity Rtx-5ms capillary column (30 m × 0.25 mm × 0.25 μm; Restek, Bellefonte, PA, USA). Five-point calibration curves, ranging from 5 to 1000 pg/μL, were used for quantification, with concentration ranges varying slightly among the different PAHs. The standard set of 17 PAHs were analyzed and quantified in triplicate (*n* = 3), and the 95% confidence interval of the concentration was calculated. The following PAHs were analyzed: naphthalene, acenaphthylene, acenaphthalene, fluorene, phenanhtrene, anthracene, fluoranthene, pyrene, benzo(a)anthracene, chrysene, benzo(b)fluoranthene, benzo(k)fluoranthene, benzo(a)pyrene, benzo(a)fluoranthene, indeno(1,2,3-c,d)pyrene, dibenzo(a,h)anthracene, and benzo(g,h,i)perylene.

### Cell cultures

The human type-II-like alveolar epithelial cell line A549 and the human bronchial epithelial cell line BEAS-2B were purchased from the American Type Tissue Culture Collection (ATCC, Rockville, MD) and maintained according to ATCC protocols. Briefly, A549 were cultured in F12 Ham medium supplemented with 10% FCS and 2 mM l-glutamine, whereas BEAS-2B were cultured in LHC-9 serum-free bronchial epithelial growth medium on noncoated plates. Both cell lines were maintained in an incubator at 37 °C with 5% CO_2_.

### Transmission electron microscopy analysis of cellular uptake of DEPs by cells

The cells were treated with 50 μg/mL of the three types of DEP for 24 h, fixed with 2.5% glutaraldehyde, post-fixed with OsO_4_, dehydrated in the graded concentrations of ethanol (Cai et al. 2007), and finally embedded in Epon. Ultra-thin sections (~80 nm) were cut and then observed using transmission electron microscope JEOL 1200 EXII (JEOL, Japan) operating at an acceleration voltage of 120 kV. To avoid the false-positive results from precipitation artifacts, sections on grids were not contrasted with uranyl acetate and lead citrate. The samples were prepared on copper mesh covered with a carbon film as carrier. Digital images were recorded by CD camera SIS Morada 11 megapixels and processed using AnalySIS.

### Flow cytometry evaluation of cellular binding/uptake kinetics of DEPs by cells

The kinetics of cellular binding/uptake of the different DEPs into BEAS-2B and A549 cells was examined by flow cytometry (Zucker et al. 2010; Lankoff et al. 2012). The approach was based on analysis of forward scatter (FSC) vs. side scatter (SSC) of measured samples. Side scatter distribution ratio was chosen as a measure of cellular uptake and was calculated by dividing the SSC value in the particle-treated cells by the SSC value in the control cells. Twenty-four hours after cell seeding, cells were incubated in six-well plates, with 1, 10, 25, 50, and 100 μg/mL of DEPs for 2, 24, and 48 h. After treatment with DEPs, cells were washed three times with PBS to remove loosely bound particles. After centrifugation, cells were resuspended in 1 mL PBS. Following gating, control and particle-exposed cells were run and plotted to examine the increase in side scatter (SSC). Data for 50,000 events per point were stored. Because the flow rate affects these measurements, they were always performed at low flow rates. The cytometer (Becton Dickinson LSR II flow cytometer equipped with 488 nm laser, FSC diode detector, and photomultiplier tube SSC detector) was set up to measure SSC logarithmically and FSC linearly.

### Analysis of apoptotic and necrotic cell death by the Annexin PI method

BEAS-2B and A549 cells at exponential growth were incubated with 1, 10, 25, 50, and 100 μg/mL of the different DEPs for 2, 24, and 48 h. Camptothecin (0.5 μM for 4 h) was used as a positive control. Analysis of apoptotic and necrotic cell death was carried out according to manufacturer instructions using Annexin V-FITC apoptosis detection Kit I (BD Pharmingen, USA). Briefly, cells were washed twice with cold PBS, and then resuspended in a 1× binding buffer at a concentration of 1 × 10^6^ cells/mL. The cell suspension (100 μL) was incubated with 5 μL of propidium iodide (PI) and 5 μL of Annexin V-FITC at room temperature for 15 min in the dark. The cells were resuspended in 400 μL of 1× binding buffer. The fluorescence was determined using a LSR II flow cytometer (Becton Dickinson). A computer system BD FACS DiVa (version 6.0, Becton Dickinson) was used for data acquisition and analysis. Data for 20,000 events per point were stored. Three different cell populations were discriminated within the cell gate: early apoptotic cells that expressed green fluorescence (Annexin+/IP−), late apoptotic/necrotic cells that were positive for both Annexin V-FITC and PI (Annexin+/IP+), and necrotic cells that expressed orange fluorescence (Annexin−/IP+).

### Measurements of cellular protein level by the sulforhodamine B assay

A549 and BEAS cells were seeded in 96-well culture plates in triplicates. The cells were allowed to grow at 37 °C in 5%. After 24 h, medium was discarded and 100 mm^3^ of DEP suspension was added to wells in four concentrations: 1, 10, 25, and 50 μg/mL in culture medium. After 2, 24, and 48 h, cells were fixed with 10% (wt/vol) trichloroacetic acid and stained for 1 h at 4 °C. The plates were washed five times with deionized water and then dried. When completely dry, 50 μL of SRB was added to each well for 20 min., and then the excess dye was removed by washing five times with 1% (vol/vol) acetic acid. The protein-bound dye was dissolved in 10 mM Tris-base solution for OD determination at 510 nm by the Fluorescent Microplate Reader Infinite F200/M200 (Tecan, USA). Protein level was calculated for each well as (OD510-treated cells/OD510 control cells) × 100%.

### Assessment of reactive oxygen species with the H_2_DCFDA

A549 and BEAS cells were seeded in 96-well black Nunclon delta plates and cultured for 24 h in a density of 10,000 cells per well in 100 mm^3^ of culture medium. After 24 h, medium was discarded and 100 mm^3^ of nanoparticle suspension was added to wells in three concentrations: 25, 50, and 100 μg/mL in culture medium for another 2 h. After incubation, 100 mm^3^ of 10 μM solution of H_2_DCFDA in HBSS was added to each well and fluorescence was read each minute in EnVision Multilabel Reader 2104 spectroflorometer for 45 min.

### RNA isolation, reverse transcription, and real-time PCR

Total RNA was extracted from cell pellets using the RNeasy Mini Kit (Qiagen) according to the manufacturer’s protocol. To assess the concentration and purity of RNA, the portion of every RNA sample was diluted in TE buffer (pH 8.0) and the absorbance at 230, 260, and 280 nm was measured using Cary 50 UV-Vis spectrophotometer (Varian). All RNA samples used in subsequent analyses had a concentration ≥100 ng/μL, as well as A260/A280 and A260/A230 ratios ≥2.0. RNA integrity was tested by agarose gel electrophoresis. For PCR array analysis, 1 μg of total RNA was converted to complementary DNA (cDNA) in a 20-μL reaction volume using RT^2^ First Strand Kit (Qiagen). The cDNA was diluted with 91 μL distilled water and used for the expression profiling using the Human Stress and Toxicity Pathway Finder PCR Array (Qiagen, cat. no. PAHS-003Z) according to the manufacturer’s instructions. Briefly, a total volume of 25 μL of PCR reaction mixture, which included 12.5 μL of RT^2^ SYBR Green/ROX qPCR Master Mix from Qiagen (containing HotStart DNA Taq polymerase, SYBR Green dye, and the ROX reference dye), 11.5 μL of double-distilled H_2_O, and 1 μL of diluted template cDNA, was used for each primer set in each well of the PCR array. One technical replicate was performed for each sample. PCR amplification was carried out using 7500 Real-Time PCR System (Thermo Fisher Scientific) with an initial 10 min step at 95 °C followed by 40 cycles of 95 °C for 15 s and 60 °C for 1 min. Relative gene expression was calculated using the ΔΔCt method with ACTB, B2M, GAPDH, HPRT1, and RPLP0 as reference controls. Calculations were done using Relative Quantification Software version 3.2.1-PRC-build1 (Thermo Fisher Cloud). Statistical differences were examined by Student’s *t* test with *p* < 0.05 considered to be statistically significant.

### Statistical evaluation

Statistical analysis of the obtained data was performed using Statistica 7.1 software (Stat Soft. Inc. Tulsa, USA). The data were expressed as mean ± standard deviation (SD) of at least three independent experiments. Data were evaluated by Kruskal-Wallis one-way analysis of variance (ANOVA) on ranks followed by the post hoc Fisher’s test. Correlation coefficients between the obtained data were evaluated by the Pearson product moment. Differences were considered statistically significant when the *p* value was less than <0.05.

## Results

### Physicochemical characterization of DEPs

**Fig. 1:**
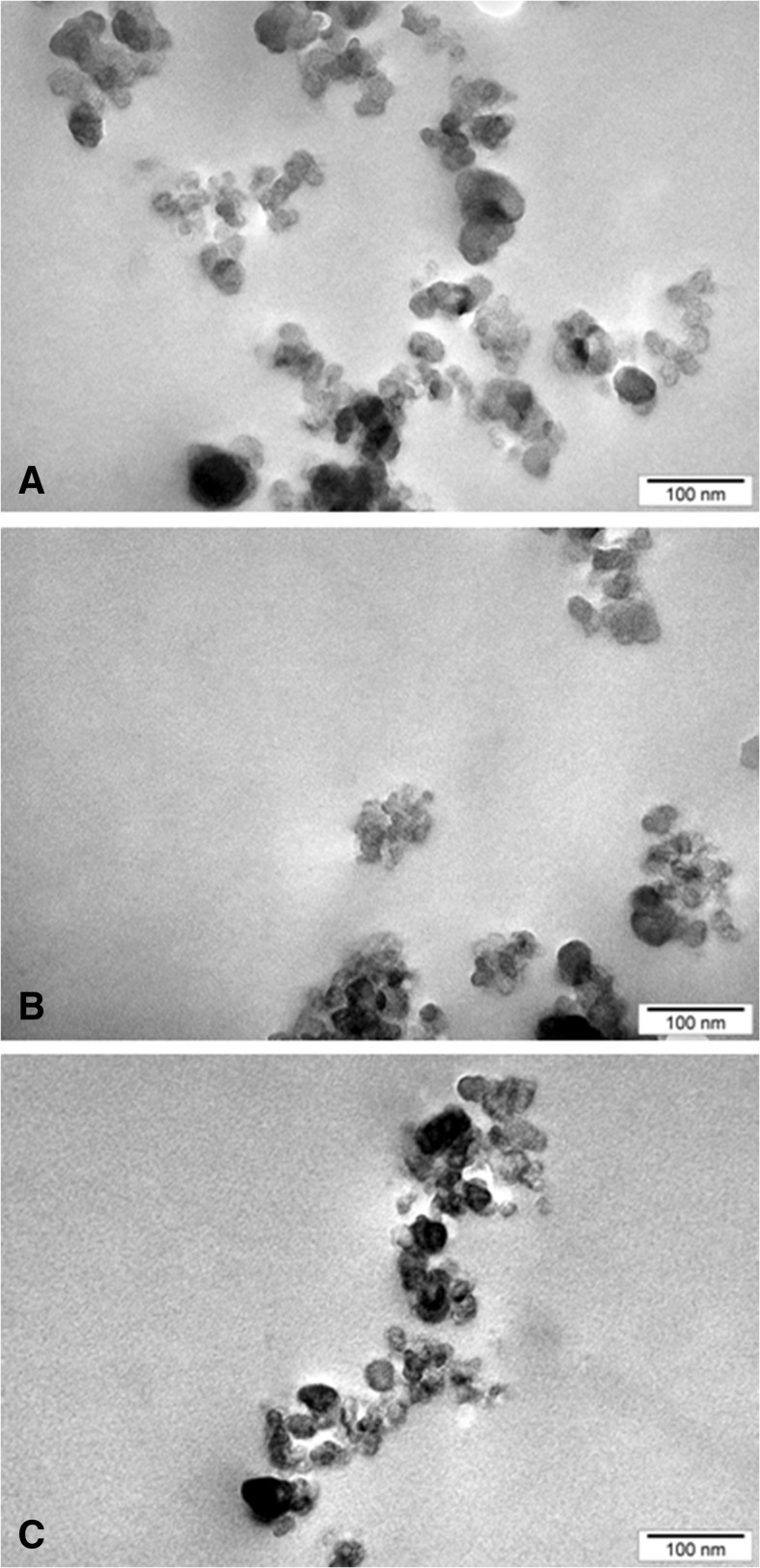
TEM images of B7-derived DEPs (**a**), B20-derived DEPs (**b**), and SHB-derived DEPs (**c**). The particles were prepared according to protocol described in “Materials and methods.” A *scale bar* corresponds to 100 nm

After dispersion of all DEP samples according to the protocol described in “Materials and methods,” the hydrodynamic size of particles was determined in various culture media by NTA measurements. As presented in Table 1, our studies revealed that the average hydrodynamic diameters of all three types of DEPs were comparable, if the same culture medium was used. About 55% of particles generated by the B7 biofuel, 70% particles generated by the B20 biofuel, and 85% particles generated by the SHB biofuel, were in the size range from 1 to 90 nm (Supplementary materials). DLS measurements showed that the polydispersity index values for all DEPs were less than 0.5, indicating high homogeneity of the suspension. Measured zeta potentials were negative and relatively similar for all tested DEPs, indicating stability of the colloidal system. DEPs were further characterized by TEM. As presented in Fig. 1, the shape of all three types of DEPs was nearly spherical. These particles formed clusters/agglomerates. However, it should be noted that these particles could agglomerate on the grid, thus observed agglomerates might be artifacts resulting from the sample preparation. The SEM-EDX analysis indicated that particles were composed primarily of carbon (Table 2). On average, the concentration of carbon in B7-DEP was 85.53%, in B20-DEP was 86.76%, and in SHB-DEP was 87.51%. The next most abundant elements were oxygen and nitrogen. Zinc was present in all DEPs at lower concentration. Concentrations of sulfur, cooper, and chlorine were below 0.5% in all samples, with exception of chlorine in SHB-DEP (1.33%). Silver and iron could only be detected in SHB-DEP samples. The samples were analyzed for 17 PAH compounds (Table 2). The total content of PAHs was the highest in the B7-DEP extract (165.78 ng/mg) and the lowest in the extracts from SHB-DEPs (69.93 ng/mg). The data showed that pyrene, fluoranthene, phenanthrene, and chrysene were the most abundant PAHs in all samples.

**Table 1:** Particle size, polydispersity, and zeta potential of B7-DEPs, B20-DEPs, and SHB-DEPs in cell culture medium (F12 + FBS and LHC-9)

Diesel exhaust particles (DEPs)	Cell culture medium	Hydrodynamic diameter^a^ (nm)	PDI	Zeta potential (mV)
B7-DEPs	F12 + FBS	78 ± 55	0.185 ± 0.02	−22.4 ± 3.22
	LHC-9	126 ± 64	0.190 ± 0.01	−21.5 ± 2.31
B20-DEPs	F12 + FBS	80 ± 43	0.455 ± 0.05	−20.1 ± 1.98
	LHC-9	107 ± 49	0.470 ± 0.08	−19.93 ± 3.98
SHB-DEPs	F12 + FBS	68 ± 37	0.334 ± 0.06	−23.5 ± 3.03
	LHC-9	113 ± 48	0.383 ± 0.02	−22.1 ± 2.98

^a^Hydrodynamic diameter determined by nanoparticle tracking analysis (NTA)

**Table 2:** Chemical constituents in diesel engine particles generated from the combustion of three types of biodiesel fuels B7-DEPs, B20-DEPs, and SHB-DEPs

Chemical constituents of the particulate samples	Diesel engine particles		
	B7-DEPs	B20-DEPs	SHB-DEPs
Elemental composition (wt%)			
Carbon	85.53 ± 0.93	86.76 ± 1.09	87.51 ± 0.86
Oxygen	7.51 ± 1.02	6.31 ± 1.21	2.24 ± 0.85
Nitrogen	5.12 ± 0.97	5.15 ± 0.87	5.11 ± 0.85
Zinc	1.40 ± 0.44	1.24 ± 0.05	2.25 ± 0.77
Sulfur	0.22 ± 0.04	0.22 ± 0.04	0.26 ± 0.07
Copper	0.18 ± 0.03	0.19 ± 0.08	0.45 ± 0.21
Chlorine	0.04 ± 0.01	0.10 ± 0.02	1.33 ± 0.36
Silver	–	–	0.72 ± 0.12
Iron	–	–	0.21 ± 0.03
Organic components (ng/mg)			
Naphthalene	3.86	7.74	3.13
Acenaphthylene	0.26	0.92	0.14
Acenaphthalene	0.11	0.19	0.07
Fluorine	0.56	1.41	0.23
Phenanhtrene	12.33	20.83	5.13
Anthracene	1.13	1.73	0.56
Fluoranthene	36.04	6.06	6.36
Pyrene	90.33	43.23	40.53
Benzo(a)anthracene	2.07	1.14	1.76
Chrysene	10.26	3.98	6.92
Benzo(b)fluoranthene	4.30	1.65	2.80
Benzo(k)fluoranthene	2.59	1.58	1.38
Benzo(a)pyrene	0.38	0.83	0.22
Benzo(a)fluoranthene	0.65	1.57	0.27
Indeno(1,2,3-c,d)pyrene	0.36	0.44	0.19
Dibenzo(a,h)anthracene	0.07	0.24	0.05
Dibenzo(g,h,i)perylene	0.47	0.33	0.20
Total PAHs	165.78	93.54	69.93

Elemental composition (wt%) with standard deviation from three independent measurements. PAH concentrations (ng/mg) in the organic extracts from B7-DEPs, B20-DEPs, and SHB-DEPs

### Cellular binding/uptake of DEPs into BEAS-2B and A549 cells

**Fig. 2:**
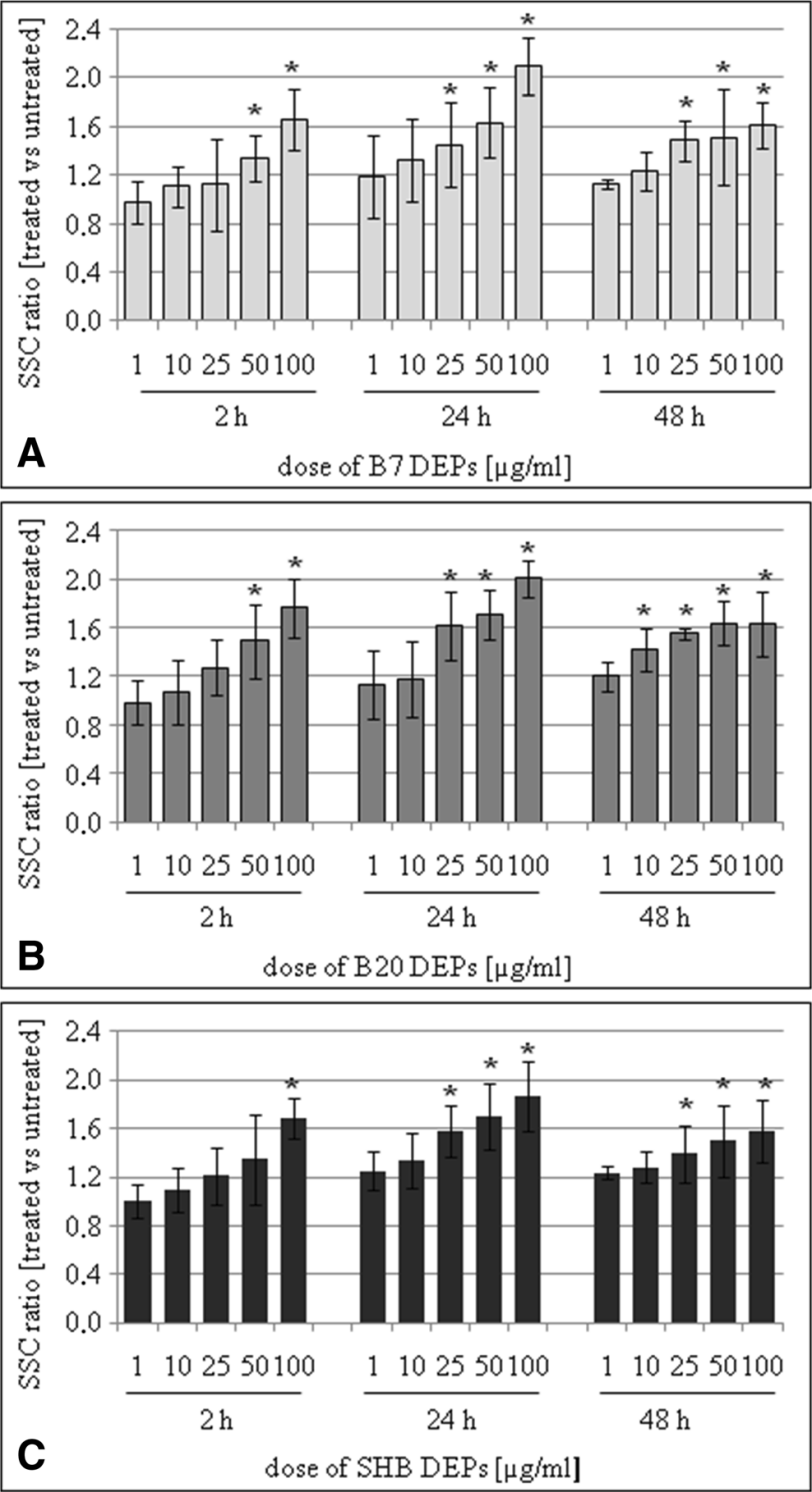
The kinetics of cellular binding/uptake of B7-derived DEPs (**a**), B20-derived DEPs (**b**), and SHB-derived DEPs (**c**) into BEAS-2B cells. The cells were treated with 1, 10, 25, 50, and 100 μg/mL of the particles for 2, 24, and 48 h. Side scatter distribution (*SSC*) ratio was chosen as a measure of cellular binding/uptake. Data are expressed as means ± SD from three independent experiments

**Fig. 3:**
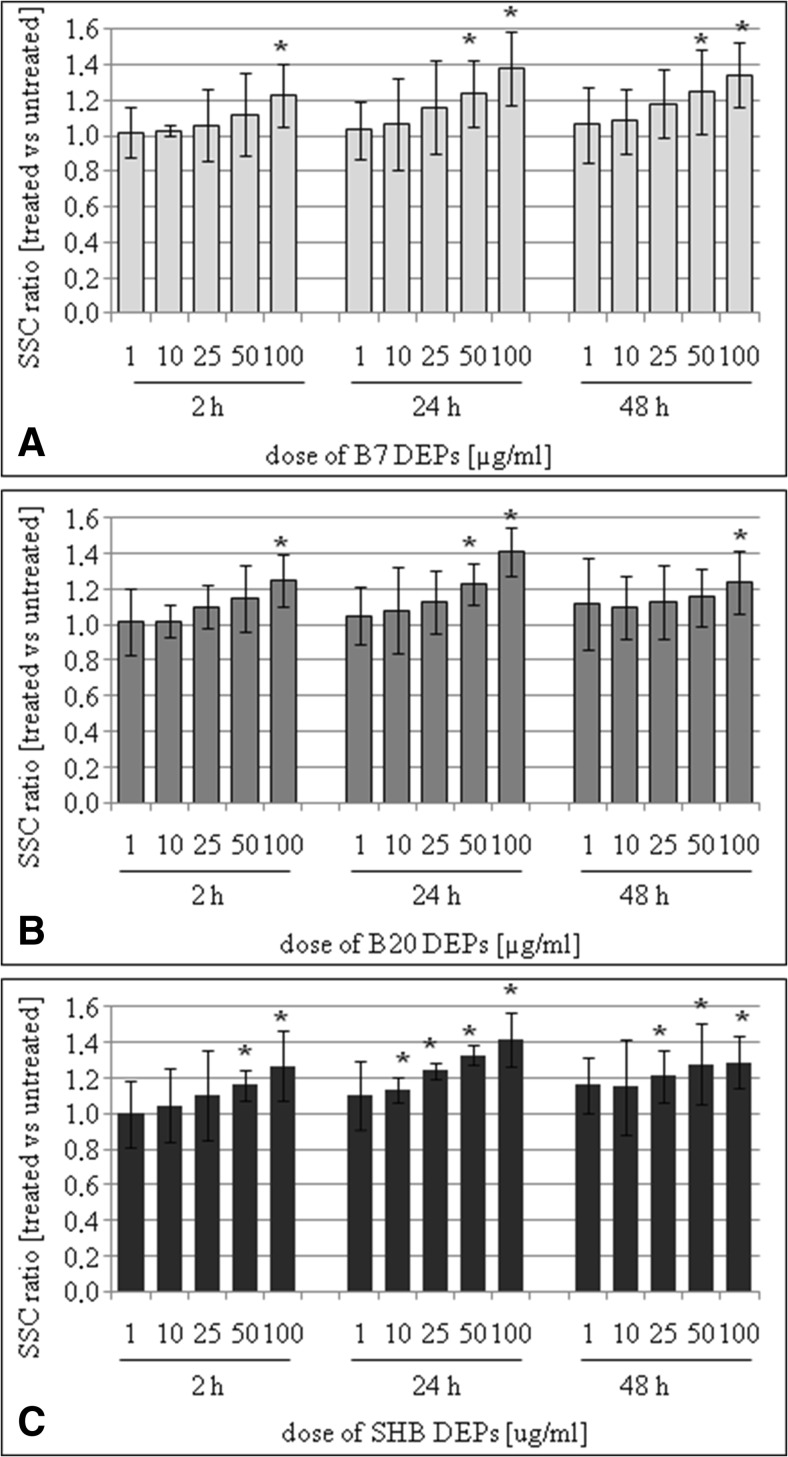
The kinetics of cellular binding/uptake of B7-derived DEPs (**a**), B20-derived DEPs (**b**), and SHB-derived DEPs (**c**) into A549 cells. The cells were treated with 1, 10, 25, 50, and 100 μg/mL of the particles for 2, 24, and 48 h. Side scatter distribution (*SSC*) ratio was chosen as a measure of cellular binding/uptake. Data are expressed as means ± SD from three independent experiments

**Fig. 4:**
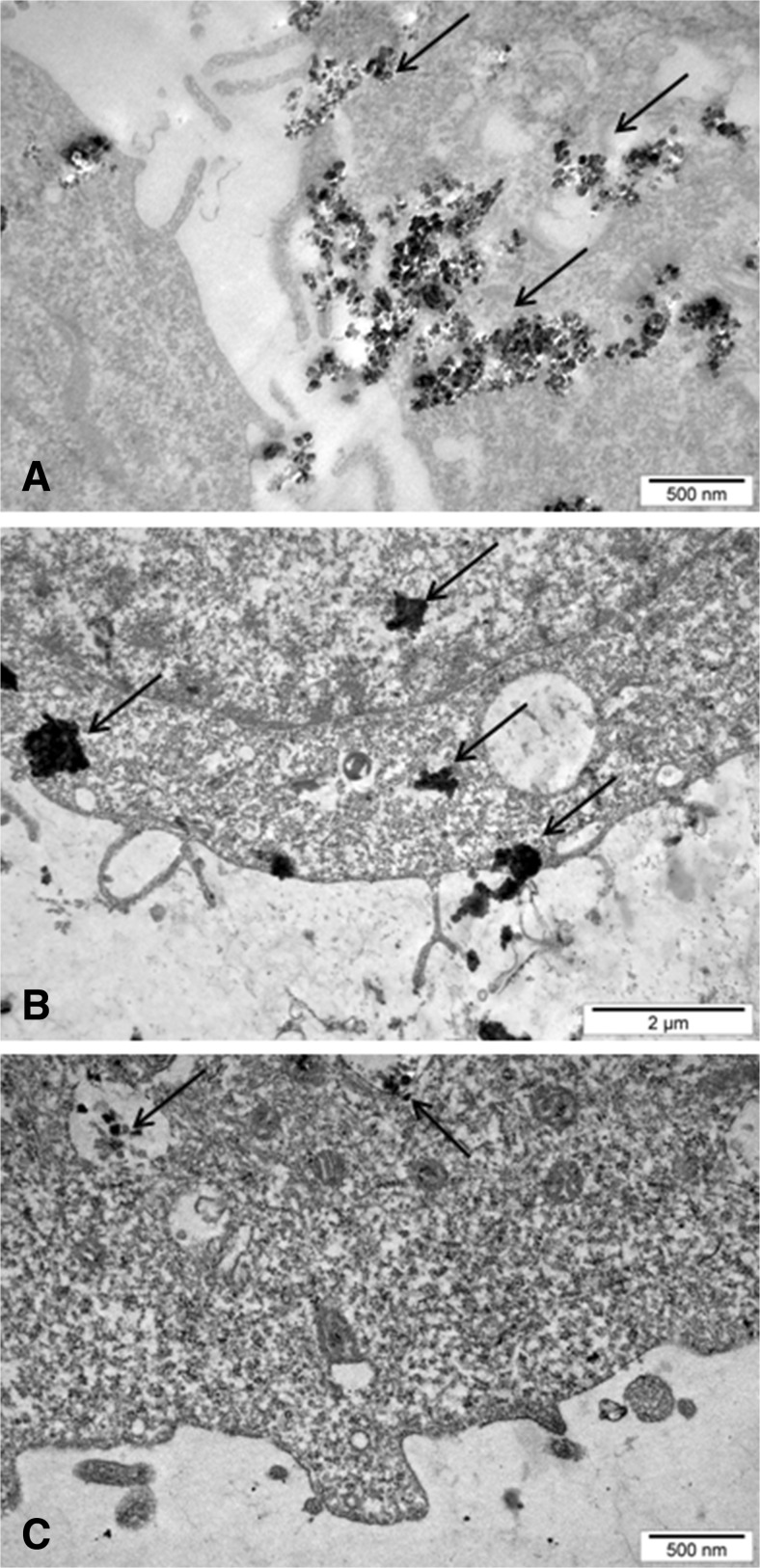
Cellular distribution of DEPs in BEAS-2B cells. The cells were exposed to 50 μg/mL of B7-derived DEPs (**a**), B20-derived DEPs (**b**), and SHB-derived DEPs (**c**) for 24 h. The *arrows* indicate NPs in the nucleus, cytoplasm, and vacuoles

The kinetics of cellular binding/uptake of DEPs into BEAS-2B and A549 cells was examined by flow cytometry. A concentration-dependent cellular binding/uptake of all DEPs into BEAS-2B cells (Fig. 2) and A549 cells (Fig. 3) was observed irrespective of types of DEP, with a progressive increase from 10 to 100 μg/mL. However, a time-dependent increase was not observed. The SSC ratio increased gradually from 2 to 24 h and further reached a plateau or decreased at 48 h depending on the type of cells, suggesting that these DEPs were removed from cells. The uptake of DEP into BEAS-2B and A549 cells was also analyzed by transmission electron microscopy. The cells were exposed to 50 μg/mL of all types of DEPs for 24 h. As presented in Fig. 4, all types of DEPs were visible mainly as large aggregates/clusters distributed in the cytoplasm and in vacuoles (Fig. 4a, c). Deposits of all DEPs were also observed in the nuclei (Fig. 4b). Compared with control cells, no visible morphological changes were detected in the BEAS-2B and A549 cells exposed to DEPs. There were no apparent differences in cellular binding/uptake kinetics between the three DEP samples.

### Induction of apoptosis and necrosis in BEAS-2B and A549 cells exposed to DEPs

**Fig. 5:**
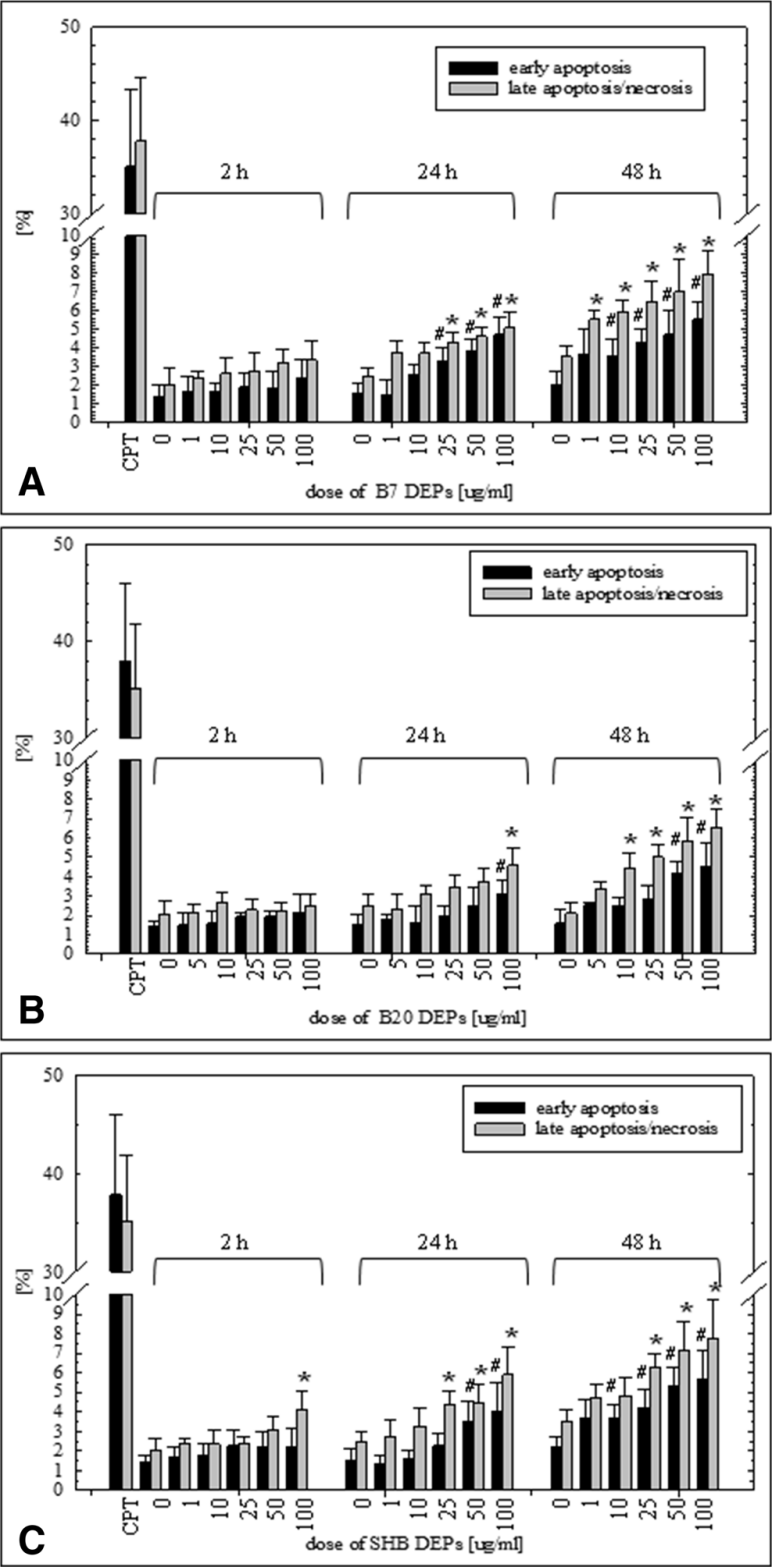
Effect of DEPs on induction of early apoptosis and late apoptosis/necrosis in BEAS-2B cells. Cell death was determined by the Annexin V-PI assay. **a** B7-derived DEPs, **b** B20-derived DEPs, and **c** SHB-derived DEPs. Data are expressed as means ± SD from three independent experiments. ^#^ *p* < 0.05, statistically significant difference vs. corresponding control group in early apoptosis; *p < 0.05, statistically significant difference vs. corresponding control group in late apoptosis/necrosis

**Fig. 6:**
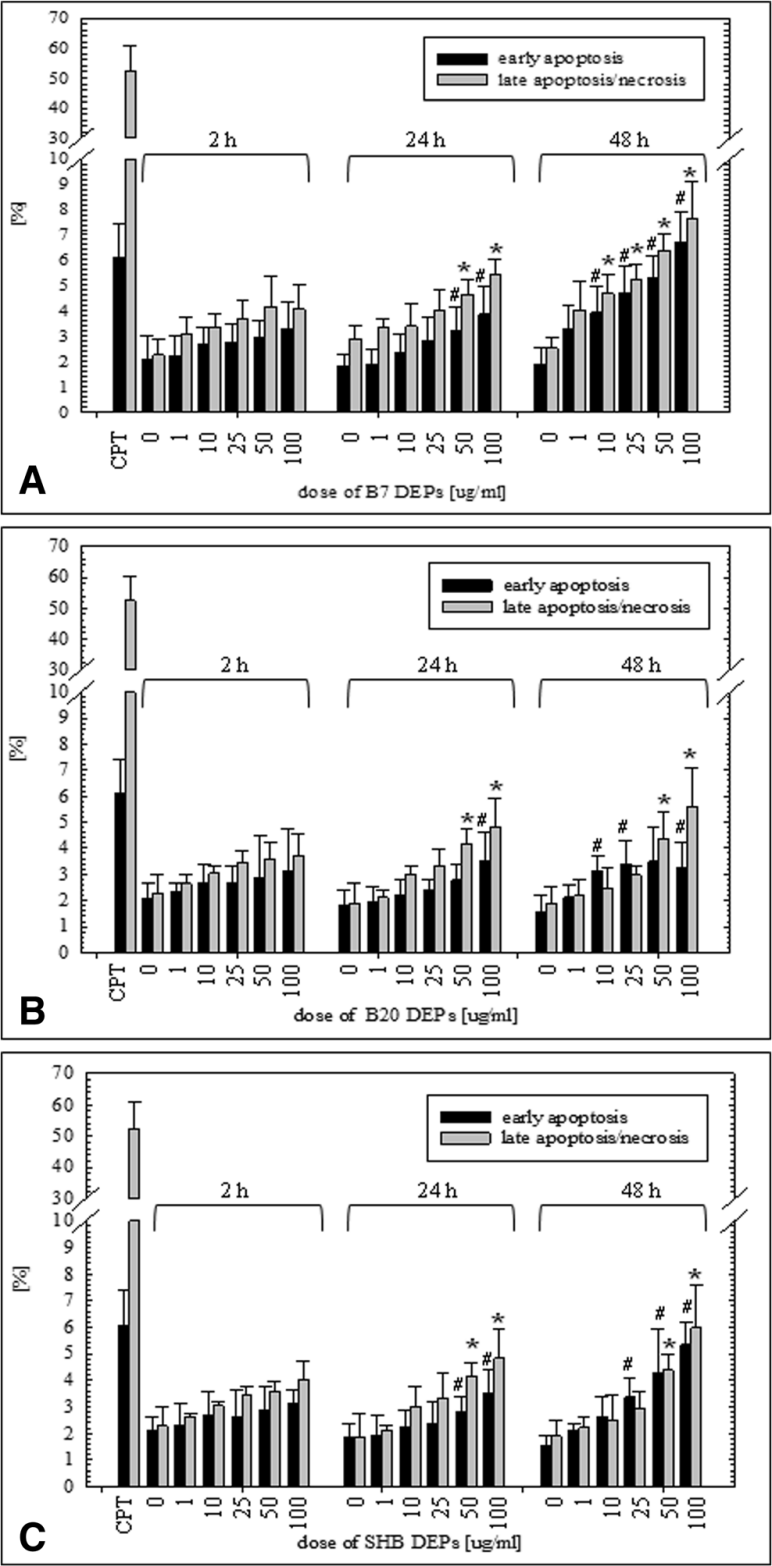
Effect of DEPs on induction of early apoptosis and late apoptosis/necrosis in A549 cells. Cell death was determined by the Annexin V-PI assay. **a** B7-derived DEPs, **b** B20-derived DEPs, and **c** SHB-derived DEPs. Data are expressed as means ± SD from three independent experiments. ^#^ *p* < 0.05, statistically significant difference vs. corresponding control group in early apoptosis; *p < 0.05, statistically significant difference vs. corresponding control group in late apoptosis/necrosis

The influence of the three types of DEPs on early apoptosis and late apoptosis/necrosis of BEAS-2B and A549 cells was measured by Annexin V-FITC/PI assay after 2-, 24-, and 48-h treatments with particles. Flow cytometric analysis revealed that all three types of DEPs increased a percent of early apoptotic and late apoptotic/necrotic cells in a dose- and time-dependent manner. As presented in Fig. 5a, B7-DEP induced early apoptosis of BEAS-2B cells at 25 μg/mL following 24-h treatment and at 10 μg/mL following 48-h treatment. Late apoptosis/necrosis was induced at 25 μg/mL following 24-h treatment and at 1 μg/mL following 48-h treatment. B20-DEP increased a percent of early apoptotic cells at 100 μg/mL following 24-h treatment and at 50 μg/mL following 48-h treatment. Late apoptosis/necrosis was induced at 100 μg/mL following 24-h treatment and at 10 μg/mL following 48-h treatment. A similar trend was observed in cells treated with SHB-DEP (Fig. 5c). Early apoptosis was induced at 50 μg/mL following 24-h treatment and at 10 μg/mL following 48-h treatment. A percent of late apoptotic/necrotic cells was increased at 100 μg/mL already following 2-h treatment, and at 25 μg/mL following 24- and 48-h treatments. In A549 cells, the cell death responses were roughly similar to the findings observed in BEAS-2B cells (Fig. 6). As shown in Fig. 6a, B7-DEP induced late apoptosis/necrosis at 50 μg/mL following 24-h treatment and at 10 μg/mL at 48-h treatment. A percent of early apoptotic cells was increased at 50 μg/mL following 24-h treatment and at 10 μg/mL following 48-h treatment. B20-DEP increased late apoptosis/necrosis at 50 μg/mL following 24-h treatment and at 50 μg/mL following 48-h treatment. Early apoptosis was elevated at 100 μg/mL following 24-h treatment and at 25 μg/mL following 48-h treatment (Fig. 6b). SHB-DEP (Fig. 6c) induced late apoptosis/necrosis at 50 μg/mL following 24 and 48-h treatment. A percent of early apoptotic cells was elevated at 100 μg/mL following 48-h treatment and at 25 μg/mL following 48-h treatment. Despite the fact that all types of DEP induced significantly cell death in BEAS-2B and A549 cells, the maximum percentage of late apoptotic/necrotic and early apoptotic cells did not exceed ~15%. B7-DEP were significantly more effective and B20-DEP the least effective in inducing cell death in BEAS-2B (*p* = 0.000001 for B7-DEP vs. B20-DEP; *p* = 0.000001 for SHB-DEP vs. B20-DEP; *p* = 0.3956 for B7-DEP vs. SHB-DEP) and A549 cells (*p* = 0.000001 for B7-DEP vs. B20-DEP; *p* = 0.4579 for SHB-DEP vs. B20-DEP; *p* = 0.000001 for B7-DEP vs. SHB-DEP).

### Cellular protein level in BEAS-2B and A549 cells exposed to DEPs

**Fig. 7:**
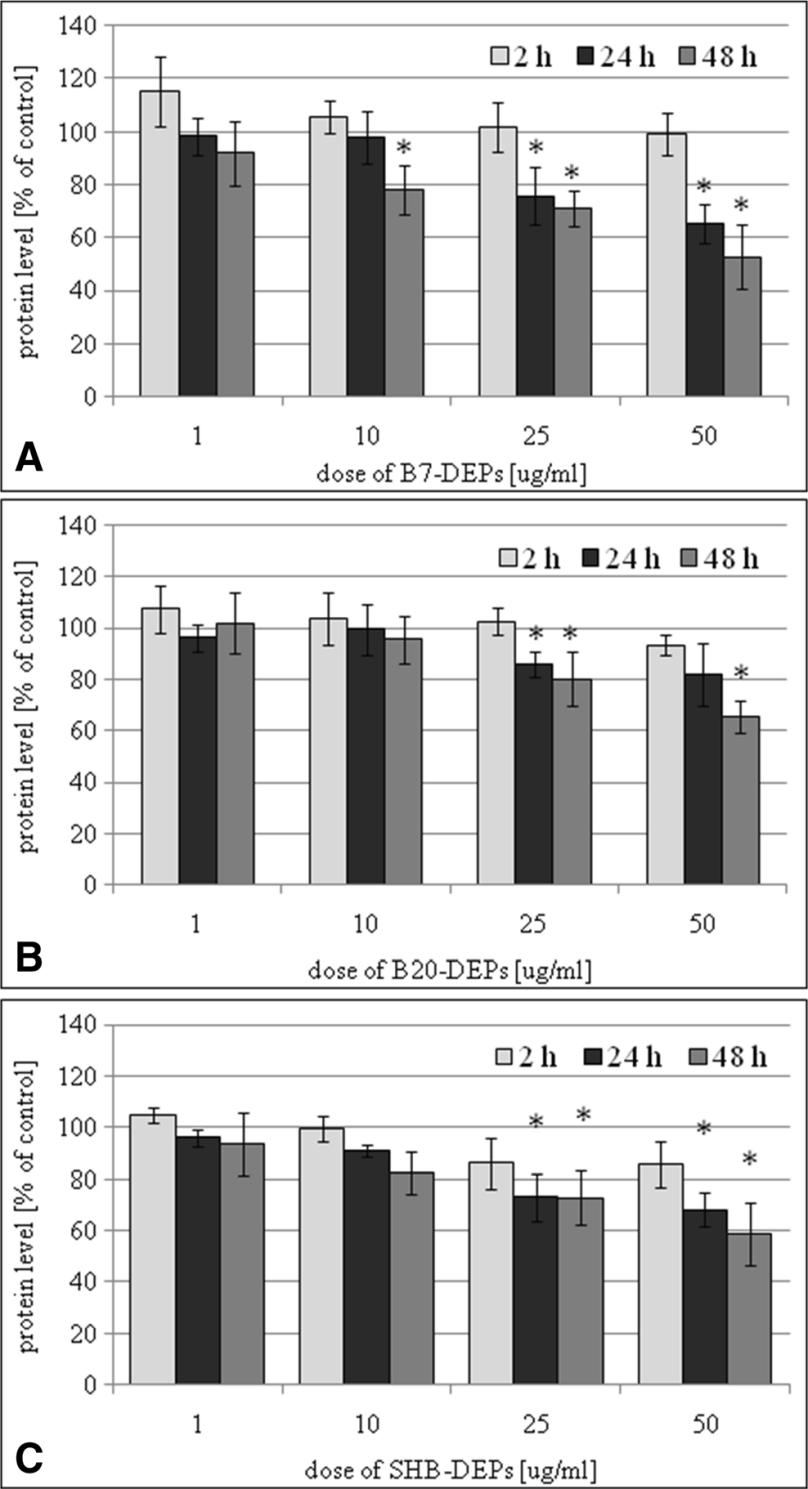
Effect of DEPs on reduction of protein level in BEAS-2B cells. The cells were treated with 1, 10, 25, and 50 μg/mL of the particles for 2, 24, and 48 h. Protein level was determined by the sulforhodamine B assay. **a** B7-derived DEPs, **b** B20-derived DEPs, and **c** SHB-derived DEPs. Data are expressed as means ± SD from three independent experiments. *p < 0.05, statistically significant difference vs. corresponding control group

**Fig. 8:**
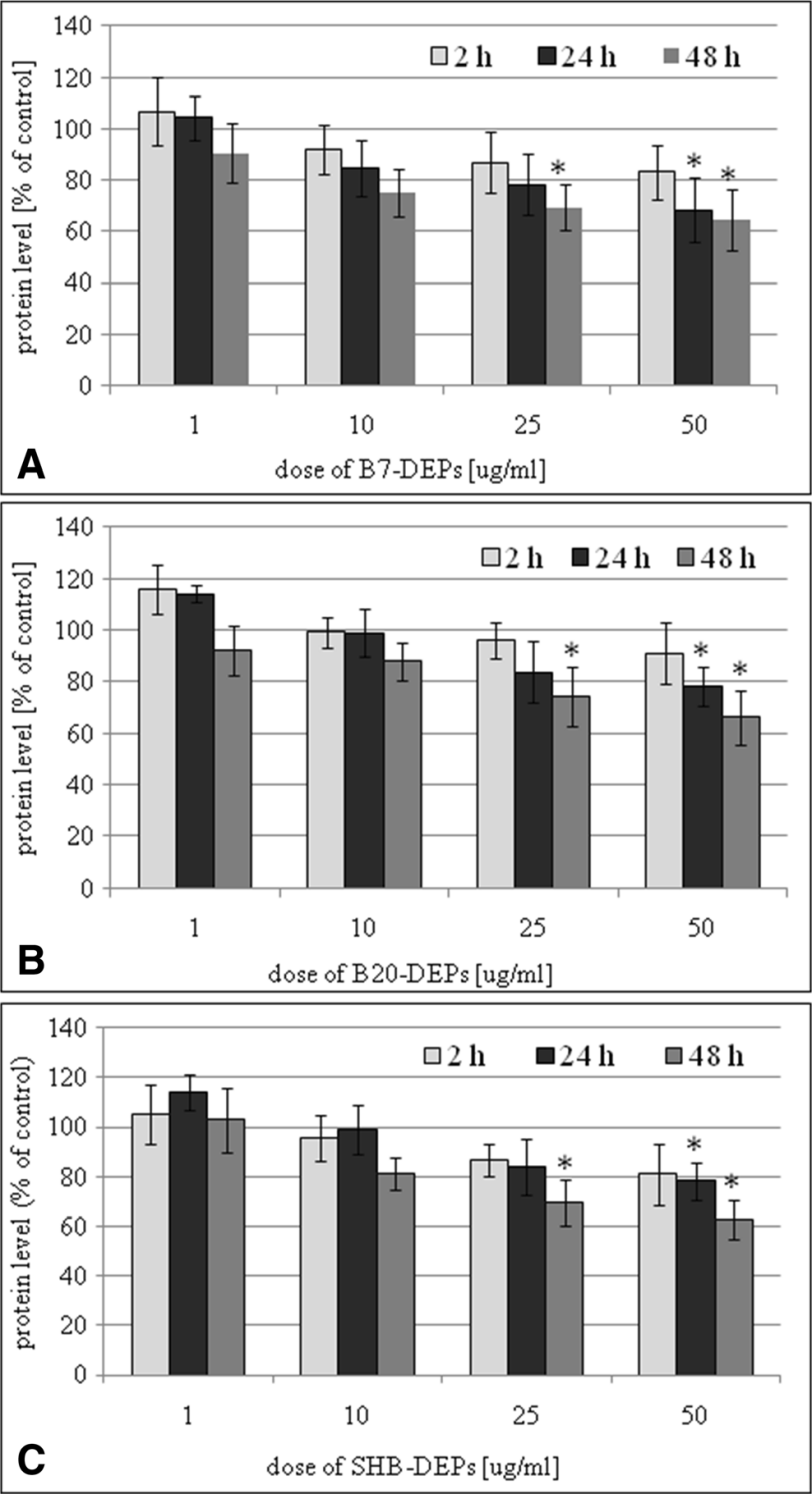
Effect of DEPs on reduction of protein level in A549 cells. The cells were treated with 1, 10, 25, and 50 μg/mL of the particles for 2, 24, and 48 h. Protein level was determined by the sulphorhodamine B assay. **a** B7-derived DEPs, **b** B20-derived DEPs, and **c** SHB-derived DEPs. Data are expressed as means ± SD from three independent experiments. *p < 0.05, statistically significant difference vs. corresponding control group

Cellular protein level in BEAS-2B and A549 cells exposed to 1, 10, 25, and 50 μg/mL of DEPs for 2, 24, and 48 h DEPs was determined by the sulforhodamine B assay. Our results presented that all three types of DEPs decreased a percent of protein level in a dose- and time-dependent manner. As presented in Fig. 7a, B7-DEP diminished a percent of protein level in BEAS-2B cells by ~25% at 25 μg/mL following 24-h treatment and by ~20% at 10 μg/mL following 48-h treatment. B20-DEP decreased a percent of protein level by ~15–20% at 25 μg/mL following 24- and 48-h treatments (Fig. 7b). SHB-DEP reduced a percent of protein level by ~25–30% at 25 μg/mL following 24- and 48-h treatments (Fig. 7c). As shown in Fig. 8a, B7-DEP diminished a percent of protein level in A549 cells by ~30% at 50 μg/mL following 24-h treatment and by ~30% at 25 μg/mL following 48-h treatment. B20-DEP decreased a percent of protein level by ~20% at 50 μg/mL following 24-h treatment and by ~25% at 25 μg/mL following 48-h treatment (Fig. 8b). SHB-DEP reduced a percent of protein level by ~30% at 50 μg/mL following 24-h treatment and by ~20% at 25 μg/mL following 48-h treatment (Fig. 8c). B7-DEP were found to be the most effective and SHB-DEP the least effective in reduction of protein level in BEAS-2B (*p* = 0.00001 for B7-DEP vs. B20-DEP; *p* = 0.00001 for SHB-DEP vs. B20-DEP; *p* = 0.00001 for B7-DEP vs. SHB-DEP) and A549 cells (*p* = 0.00001 for B7-DEP vs. B20-DEP; *p* = 0.0905 for SHB-DEP vs. B20-DEP; *p* = 0.00001 for B7-DEP vs. SHB-DEP).

### Generation of reactive oxygen species by DEPs in BEAS-2B and A549 cells

Generation of ROS in DEP-exposed BEAS-2B and A549 cells was determined by analysis of the kinetics of ROS reaction with H_2_DCFDA. Our results revealed that all DEPs significantly influenced the constant rates of dye fluorescence, indicating a dose- and time-dependent increase of the produced ROS (Table 3). B7-DEP and B20-DEP induced almost identical increases in H_2_DCFDA fluorescence, statistically significantly different from the control from 25 μg/cm^3^, while SHB only induced a significant increase from 50 μg/cm^3^ in BEAS-2B cells. However, the relative increase as compared with control, at the higher concentrations was marginally different in the BEAS-2B cells (Table 4). In the A549 cells, B7-DEP and B20-DEP again increased H_2_DCFDA fluorescence significantly from 25 μg/cm^3^. However, while B20-DEP-induced ROS production appeared to continue at higher doses, the B7-DEP response peaked at the lowest dose, and significance was lost at 100 μg/cm^3^. Moreover, SHB reduced H_2_DCFDA fluorescence significantly at 25 and 50μg/cm^3^ but not at 100 μg/cm^3^.

**Table 3:** Assessment of oxidative properties of studied DEPs with H_2_DCFDA

	BEAS-2B					
	Concentration (μg/cm^3^)	Rate constant K (1/min)	Standard error	Doubling time (ln2/K) (min)	95% confidence intervals	Degrees of freedom
B7-DEPs	100	0.06242*	0.001123	11.10	10.73 to 11.51	135
	50	0.05974*	0.0009490	11.60	11.25 to 11.97	135
	25	0.05791*	0.0009175	11.97	11.61 to 12.35	135
	0	0.05478	0.0005984	12.65	12.39 to 12.93	270
B20-DEPs	100	0.06296*	0.001051	11.01	10.66 to 11.38	135
	50	0.06031*	0.001007	11.49	11.13 to 11.88	135
	25	0.05847*	0.0009707	11.86	11.48 to 12.25	135
	0	0.05478	0.0005984	12.65	12.39 to 12.93	270
SHB-DEPs	100	0.05618*	0.001021	12.34	11.91 to 12.79	270
	50	0.05286*	0.001017	13.11	12.64 to 13.63	270
	25	0.05111	0.0008011	13.56	13.16 to 13.99	270
	0	0.04859	0.001152	14.26	13.63 to 14.96	270

BEAS-2B cells were cultured with nanoparticles in three concentrations: 100, 50, and 25 μg/cm^3^ for 2 h. Fluorescence was read each minute for 45 min. Data points were fit to exponential growth equation (*y* = *y*_0_ ⋅ *e* ^*K* ⋅ *x*^)

*Difference towards respective control

**Table 4:** Assessment of oxidative properties of studied DEPs with H_2_DCFDA

	A549					
	Concentration (μg/cm^3^)	Rate constant K (1/min)	Standard error	Doubling time (ln2/K) (min)	95% confidence intervals	Degrees of freedom
B7-DEPs	100	0.04138	0.0002685	16.75	16.54 to 16.97	271
	50	0.04479*	0.0004004	15.48	15.21 to 15.75	271
	25	0.04650*	0.0006699	14.91	14.50 to 15.34	271
	0	0.04100	0.0005522	16.91	16.47 to 17.36	273
B20-DEPs	100	0.04557*	0.0004950	15.21	14.89 to 15.54	271
	50	0.03980*	0.0004198	17.41	17.06 to 17.78	271
	25	0.03750*	0.0004537	18.48	18.05 to 18.93	226
	0	0.03445	0.0005176	18.15	17.51 to 18.83	271
SHB-DEPs	100	0.03658	0.0007146	18.95	18.25 to 19.70	271
	50	0.03221*	0.001052	21.52	20.23 to 22.99	271
	25	0.03121*	0.0009429	22.21	20.97 to 23.61	271
	0	0.03820	0.0007101	18.15	17.51 to 18.83	271

A549 cells were cultured with nanoparticles in three concentrations: 100, 50, and 25 μg/cm^3^ for 2 h. Fluorescence was read each minute for 45 min. Data points were fit to exponential growth equation (*y* = *y*_0_ ∙ *e* ^*K* ∙ *x*^)

*Difference towards respective control

### Gene expression profiling in BEAS-2B and A549 cells exposed to DEPs

**Fig. 9:**
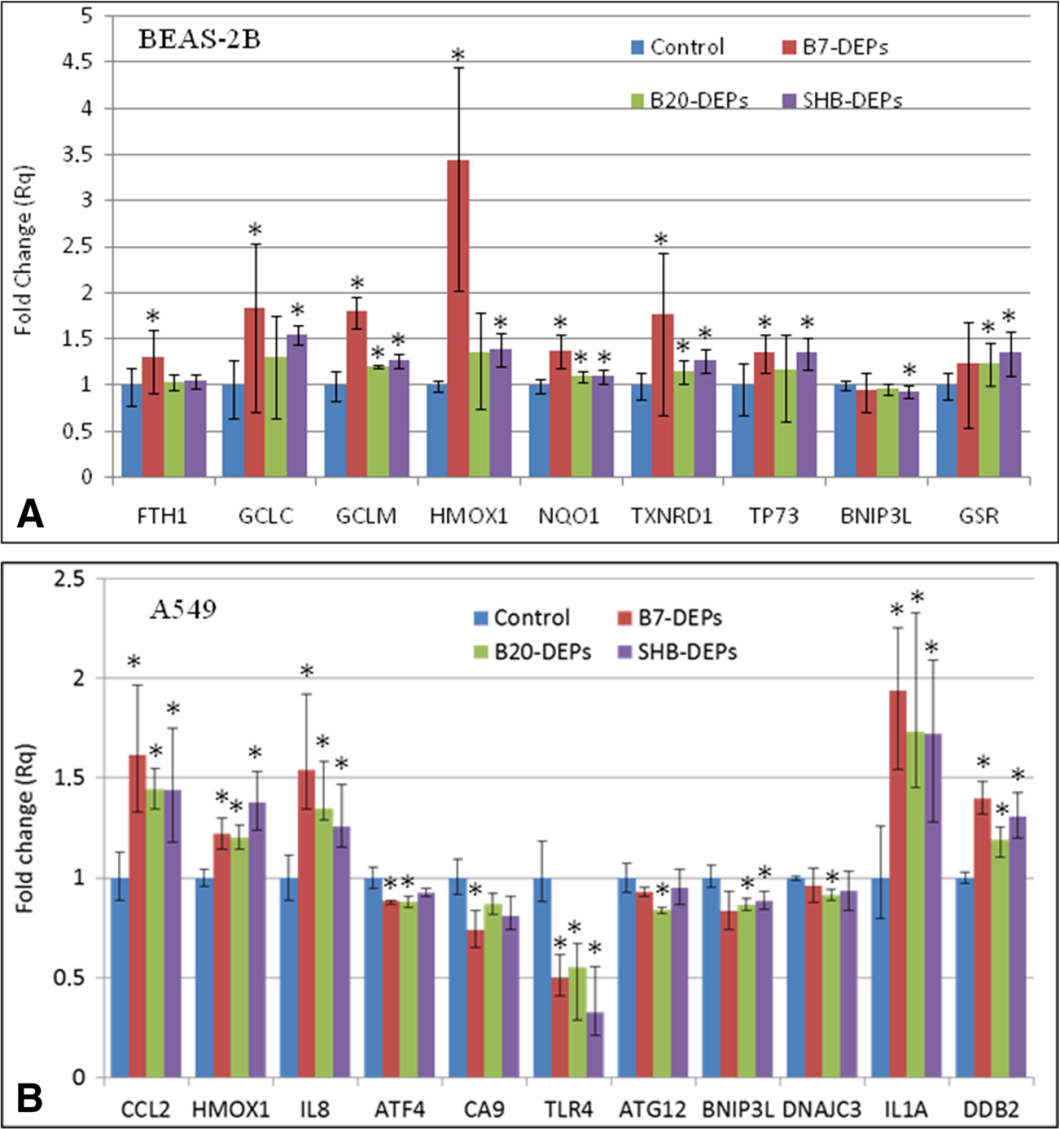
Changes in gene expression in BEAS-2B cells (**a**) and A549 cells (**b**) after treatment with 50 μg/mL of three types of DEPs for 6 h. Mean fold-change values from three independent experiments are presented. *Error bars* represent minimum and maximum values in a sample. Fold changes statistically significant in Student’s *t* test are highlighted (*asterisk*)

The DEPs were analyzed by the real-time PCR for their effects on the expression of 84 different genes, regulated during cellular responses to stress and toxic compounds (Supplementary Table 1 and Table 2). These genes are related to responses to oxidative stress, cell death, osmotic stress, hypoxia signaling, inflammatory response, DNA damage and unfolded protein response. Experiments with BEAS-2B cells revealed that 10 genes appeared to be significantly deregulated by B7-DEPs (upregulated: ferritin heavy polypeptide 1 (FTH1), glutamate cysteine ligase catalytic subunit (GCLC), glutamate cysteine ligase modifier subunit (GCLM), heme oxygenase 1 (HMOX1), quinone oxidoreductase (NQO1), sequestosome 1 (SQSTM1), thioredoxin reductase 1 (TXNRD1); downregulated: IL1B, SLC5A3, TLR4), 5 genes by B20-DEPs (upregulated: GCLM, glutathione reductase (GSR), NQO1, TXNRD1; downregulated: VEGFA), and 11 genes by SHB-DEPs (upregulated: GCLC, GCLM, GSR, HMOX1, NQO1, TXNRD1; downregulated: BCL2 interacting protein 3 like (BNIP3L), DnaJ (Hsp40) homolog, subfamily C (DNAJC3), HSP90B1, SERPINE1, VEGFA) (Fig. 9a). Experiments with A549 cells revealed that 9 genes were significantly deregulated by B7-DEP (upregulated: chemokine (C–C motif) ligand 2 (CCL2), CHEK1, HMOX1, IL-8, IL1A, damage-specific DNA binding protein 2 (DDB2); downregulated: activating transcription factor (ATF4), CA9, TLR4), 10 genes by B20-DEP (upregulated: CCL2, HMOX1, IL-8, IL1A, DDB2; downregulated: ATF4, autophagy-related protein 12 (ATG12), BNIP3L, DNAJC3, TLR4), and 7 genes by SHB-DEP (upregulated: CCL2, DDB2, HMOX1, IL-8, IL1A; downregulated: BNIP3L, TLR4) (Fig. 9b).

## Discussion

In the present study, we aimed to compare the toxicity of DEPs from the combustion of 1st- and 2nd-generation biodiesel fuels in relation to their physicochemical properties. We tested 1st-generation B7 biofuel, which is currently used in Europe (7% FAME in diesel oil), 1st-generation B20 biofuel (20% FAME in diesel oil) and 2nd-generation SHB biofuel (7% FAME and 13% synthetic HVO). These biofuels were combusted under identical engine operation conditions; emissions were evaluated during a certified test cycle and comprised measurements of regulated (not shown) and nonregulated emissions. Our results revealed that the average hydrodynamic diameters of all three types of DEPs were comparable. However, the B7 biofuel generated the lowest number of particles with diameters in the range 1–90 nm (~55%) as compared with the B20 biofuel (~70%) and the SHB biofuel (~85%). These findings are in line with Surawski et al. (2011), who reported that a decrease in particle emissions with increasing blend percentage was followed by emission of smaller particles. In addition, reduction of particulate matter with increasing proportion of biocomponent was shown by others (Betha and Balasubramanian 2013; Bünger et al. 2012; Libalova et al. 2016). In contrast, Steiner et al. (2013) reported that the combustion of B100 (100% RME) resulted in the formation of three times more particles than B20 (20% RME in diesel oil) and B0 (100% pure diesel). While conducting the physicochemical characterization, we observed that the shape of all three types of DEPs was nearly spherical. The particles were present as a single particles, clusters or chain-like aggregates, which are usually described as soot (La Rocca et al. 2015). The soot observed in this study was similar to typical aggregates/agglomerates from diesel engines (Braisher et al. 2010; Uy et al. 2014).

Beside the quantity and size of emitted particles, their chemical composition also influences their toxicity. The chemical composition of DEP depends mainly on engine operation conditions, type of fuel, and contaminations (Lin et al. 2005; McDonald et al. 2011; Popovicheva et al. 2015). It therefore seemed interesting to analyze influence of different biofuels on elemental composition of particles generated in our study. These results showed that all DEPs were composed primarily of carbon (~85%). The next most abundant elements were oxygen and nitrogen. Zinc was present in all DEPs at lower concentration. Concentrations of sulfur, cooper, and chlorine were below 0.5% in all samples, with exception of chlorine, which were slightly above 1% in SHB-DEP. Silver and iron could only be detected in SHB-DEP samples. A comparative analysis of elements among all DEPs samples showed the similar elemental profile for B7-DEP and B20-DEP. However, elemental profile for SHB-DEP varied significantly, showing higher concentrations of transition metals (iron, zinc and copper) and presence of silver. Our results are in accordance with previous research showing that the composition of carbonaceous biodiesel particles varies in a wide concentration range of trace elements and results from the composition of biodiesel fuel and residual amount of chemicals used as a catalysts during biodiesel production (Betha and Balasubramanian 2013; Popovicheva et al. 2015).

The three DEP samples were also analyzed for levels of 17 PAH compounds, including PAHs classified by the International Agency for Research on Cancer (IARC) as carcinogenic or probably/possibly carcinogenic to humans. The total content of PAHs was the highest in the extract from B7-DEP, lower in the extract from B20-DEP and the lowest in the extract from SHB-DEP. Among these 17 PAHs, pyrene, fluoranthene, phenanthrene, and chrysene were the most abundant in all DEP samples. In general, the PAH profiles determined in this study resemble those found in the literature (Song et al. 2011; Bakeas and Karavalakis 2013; Vojtisek-Lom et al. 2015). However, the results from our and others studies show no consistent pattern in regards to the particular blend (Bünger et al. 2012). In the study of Ratcliff et al. (2010), soy-based biodiesel at 100% (B100) and 20% (B20) blends reduced engine-out emissions of PAHs, compared with pure diesel (B0). In contrary, Libalova et al. (2016) reported that the concentration of PAHs in organic extracts was elevated with increasing proportion of the RME blending ratio (B30 or B100), compared with pure diesel (B0), but was the lowest for 100% HVO (NEXTBTL).

To relate the chemical and physical properties of the DEPs to their biological effects, we investigated how the diversity in composition of three types of DEP affected their uptake into cells. In previous in vitro studies, cellular uptake of DEP have been reported in human alveolar macrophages, human dendritic, bronchial and alveolar epithelial cells, as well as triple cell co-cultures (Reibman et al. 2002; Beck-Speier et al. 2005; Müller et al. 2016). We used BEAS-2B (immortalized human bronchial epithelial) and A549 (adenocarcinomic human alveolar basal epithelial) cells, derived from the respiratory tract, which is the primary route of exposure to inhaled DEPs. Our results revealed a concentration-dependent cellular binding/uptake of all DEPs irrespective of types of DEPs. The cellular binding/uptake of particles increased gradually from 2 to 24 h and further reached a plateau or decreased at 48 h, depending on the type of cells. As it was not possible to determine by flow cytometry whether these DEPs were inside the cells or whether they were attached to the cell surface, we verified this phenomenon by transmission electron microscopy. We found that all types of DEPs were visible mainly as large aggregates/clusters, distributed in the cytoplasm and in vacuoles. Deposits of DEPs were also observed in the nuclei. These findings were independent on the type of DEPs, confirming the flow cytometry data.

Once it had been shown that all DEPs were efficiently taken-up by cells, we compared the potential of the particles to induce the cell death. In addition Annexin V-FITC assay combined with a vital staining—propidium iodide—was used to determine the mode of death. The assay allows to quantify translocation of phosphatidylserine from the inner to the outer leaflet of the plasma membrane, reflecting the earliest manifestations of apoptosis, and loss of membrane integrity, indicating late apoptosis/necrosis (Wlodkowic et al. 2011). Our results revealed that all three types of DEPs increased the percentage of early apoptotic and late apoptotic/necrotic cells in a dose- and time-dependent manner. However, the overall percentage of death cells did not exceed ~15% at the highest dose following 48-h treatment time. This indicates that the acute cytotoxic potential of the three DEPs were low, which is in agreement with other reports (Liu et al. 2008; Swanson et al. 2009; Steiner et al. 2013). Regardless of the relatively low cytotoxicity of all studied DEPs, B7-DEP was found to be more effective in inducing cell death than B20-DEP and SHB-DEP, indicating that the acute toxicity decreases with the increased concentration of biocomponent. Interestingly, DEPs from combustion of SHB20 appeared to be more toxic than DEPs from B20, despite that both biofuels contain the equal concentration of biocomponent. This suggests that apart from the proportion of biocomponent in the diesel oil, also chemical character of the component is an important factor affecting DEP-induced cell death. Our results are in accordance with previous research showing that addition of increasing amounts of DEPs leads to a dose-dependent increase in the percentage of apoptotic and necrotic RAW 264.7, THP-1 and BEAS-2B cells, and that the chemical composition of carbonaceous biodiesel particles is responsible for initiating the acute toxicity (Hiura et al. 1999).

To further explore the mechanisms of toxicity of DEPs, we evaluated the cellular protein concentration in living BEAS-2B and A549 cells by the sulforhodamine B (SRB) assay. This assay is widely described as a “cytotoxicity assay” (Vichai and Kirtikara 2006). However, the observed decrease in cell protein concentration may result not only from cell death but also from a combination of alterations in cell biochemistry and anti-proliferative effects (Blois et al. 2011). Our results showed that 48-h treatment with each of the three DEP samples decreased cellular protein content significantly (~40%) at the highest dose. B7-DEP appeared to be the most effective and SHB20-DEP were more effective than B20-DEP in reduction of cellular protein content. The lack of correspondence between the SRB results (~50%) and the Annexin PI assay results (~15%) suggests that beside the induction of cell death, DEPs can also slow down the cellular doubling time, likely interfering with cell cycle progression and inducing growth arrest.

Formation of ROS and subsequent oxidative stress has been a central paradigm for the toxicity of DEP and other particlulates (Øvrevik et al. 2015). At present, the impact of different biodiesel types and blend percentages on cellular ROS formation and oxidative stress responses remains unclear. Our results showed that all types of DEPs significantly affected the rate constants of increase of HDCF fluorescence, indicating a dose- and time-dependent increase of the produced ROS. The fluorescent probe (HDCF) used in this assay reacts towards a broad spectrum of ROS, including hydroxyl radicals, peroxide, superoxide radicals, and peroxynitrite, and allows for comprehensive assessment of the total DEPs oxidative activity generated within the cell (Carranza and Pantano 2003). We found that all three types of DEPs induced significantly ROS in BEAS-2B, showing no considerable differences between DEPs. Whereas, the ROS production in A549 was more varied, with SHB-DEP inducing a reduction in ROS. Comparing our results to the literature, partially similar results were reported by Jalava et al. (2010), who found that organic extracts from pure diesel and biodiesel (RME) particles induced a concentration-related generation of ROS and extracts from RME particles induced significantly more ROS than the extracts from pure diesel particles. However, Surawski et al. (2011) reported that relative to pure diesel particles, B20 tallow particles induced less ROS, but B80 tallow and B20 and B80 canola particles induced more ROS, showing that there is no strong feedstock variability. In contrast, Li et al. (2002) and Libalova et al. (2016) reported no significant increase in generation of ROS by organic extracts from pure diesel, 100% biodiesel (NextBTL), 100% biodiesel (RME), and a blend of 30% biodiesel (RME) particles. The observed inconsistency is certainly due not only to the different fuel types, engines, and engine operation conditions used in these studies but also to the various experimental approaches that have been used.

An increase in the ROS levels perturbs cellular redox balance and often leads to activation of different cellular signaling pathways and deregulation of genes encoding regulatory transcription factors, antioxidant defense enzymes, and structural protein (Dalton et al. 1999). To further explore the mechanisms of the different oxidative response of BEAS-2B and A549 cells to three types of DEPs, we analyzed the expression of 84 genes regulated during cellular response to stress and toxic compounds. Supplementary Table 1 summarizes the differential expression of the genes tested in BEAS-2B cells. Among these genes, commonly upregulated genes were GCLC, GCLM, GSR, HMOX1, TXNRD1, NQO1, FTH1, and SQSTM1—controlled by the Nrf-2 signaling pathway, which is considered to be a major cellular defense mechanism against oxidative stress (Ma et al. 2014). Elevated expressions of GCLC and GCLM may be interpreted as an antioxidant response, since these are associated with synthesis of glutathione (GSH), the principal determinant of the intracellular redox state (Weldy et al. 2011). Similarly, upregulated expression of GSR is an indicator of antioxidant responses as GSR plays a critical role in the antioxidant defense not only by reducing oxidized glutathione (GSSG) but also by clearance of electrophilic metabolite (Drozd et al. 2016). Further signs of antioxidant responses include overexpression of HMOX1, which is considered one of the most sensitive and reliable indicators of cellular oxidative stress (Maamoun et al. 2016), TXNRD1, a pivotal intracellular redox sensor and antioxidant enzyme (Schmidt 2015), NQO1, which is essential for cell defense against ROS (Di Francesco et al. 2016), and FTH1, a major iron storage protein that can protect cells from the toxic effects of iron as well as other free radical producing stresses (Funauchi et al. 2015). In addition, downregulation of the pro-apoptotic BNIP3L, which plays an important role in hypoxia-dependent cell death (Bellot et al. 2009) and upregulation of SQSTM1, a multifunctional adapter protein that accumulates following autophagy inhibition (Duleh et al. 2016), was also observed. This could explain the relatively moderate impact of the tested DEPs on cell cytotoxicity. Interestingly, we identified several genes in A549 cells that were differentially expressed as compared with BEAS-2B cells (Supplementary Table 2). Among these, there were only two genes controlled by the Nrf-2 signaling pathway: HMOX1 and GCLM in A549 cells (vs. eight genes in BEAS-2B). This result is consistent with our previous observations and existing knowledge, indicating that A549 cells are less sensitive to stress conditions than BEAS-2B cells due to mutations in the NRF2/KEAP1 pathway, active in the oxidative stress response (Lankoff et al. 2012; Brzóska et al. 2015). Instead of NRF2-regulated genes, commonly upregulated genes were CCL2, CXCL8, CHEK1, and DDB2. Overexpression of the chemokines CCL2 and CXCL8 is indicative of pro-inflammatory processes. CCL2 display chemotactic activity for monocytes and memory cells, while CXCL8 (interleukin 8) is a potent neutrophil attracting chemokine and one of the major mediators of the inflammatory response (Boshtam et al. 2016). Upregulation of CHEK1 and DDB2 may be interpreted as a response to DNA damage. CHEK1, which codes for checkpoint kinase 1 (Chk1), is involved in suppression of DNA synthesis and cell cycle progression following DNA damage (Kemp et al. 2016), while DDB2 participates in nucleotide excision repair, a DNA repair pathway involved in the removal of bulky DNA adducts (Forestier et al. 2015). In addition, the three DEPs affected expression of genes involved in hypoxia-induced cell death in A549 cells. The most commonly downregulated genes were BNIP3L, ATF4, a transcription factor playing a key role in the regulation of autophagy and apoptosis in response to severe hypoxia and ER stress (Rzymski et al. 2010); ATG12, which promotes autophagy and apoptosis through an interaction with anti-apoptotic members of the Bcl-2 family (Cufí et al. 2012); and DNAJC3, which is an important apoptotic constituent mediating translational block and is considered as marker for ER stress (Plaisance et al. 2016). Direct comparison between our current gene expression data and other studies is difficult due to differences in fuel composition. Recently, a comprehensive by Libalova et al. (2016) tested organic extracts from various diesel exhaust particles rather than exhaust particles. These authors reported that the deregulated genes included those involved in antioxidant defense, cell cycle regulation, and proliferation, similarly as in our study. However, contrary to our studies, they found that the number of deregulated genes increased with increasing ratio of biocomponent in the fuel.

Our present results underscores that the toxicity of DEPs depends both on the biodiesel blend percentage and on the biodiesel feedstock (i.e., FAME vs. HVO). This is most likely due to differences in the physicochemical properties of the three types of DEP tested. A number of characteristic parameters of particles affect their toxicity, including their size, shape, surface reactivity, surface charge, surface coating, and elemental composition (Øvrevik et al. 2015). While the size, morphology, and surface charge of three types of DEPs were similar, the presence of organic compounds including PAHs, elemental composition, and soluble toxic components on the particle surface, such as metal ions, could be expected to have an effect. B7-derived DEP, containing the highest concentration of PAHs, was most effective in inducing necrosis and apoptosis, as well as decreasing protein content in BEAS-2B and A549 cells. The increase in bioadditive ratio caused a decrease in the PAH concentration of sufficient magnitude to diminish the observed effects, as demonstrated for B20-DEP and SHB-DEPs. The role of PAHs adsorbed on diesel soot emissions has been extensively reviewed, showing that combustion particles carrying organic chemicals may clearly induce ROS formation through redox cycling of quinone species and metabolic degradation of PAHs, which has been linked to oxidative damage on macromolecules and deregulation of gene expressions (Schwarze et al. 2013). However, higher toxicity of SHB-DEP as compared with B20-DEP, may be attributed to responses triggered by soluble constituents leaking from these particles (e.g., silver ions) and presence of redox-active transition metals such as iron and copper might undergo Fenton or Haber-Weiss reactions. It was reported that particle-associated transition metals are capable of catalyzing the formation of reactive oxygen species and that they are at least in part responsible for the biological effects of DEPs (Verma et al. 2014). In addition, higher toxicity of SHB-DEP may be related to the higher number of nanoparticles with diameters in the range from 10 to 90 nm, since it is well known that small particles are generally more toxic than larger particles due to a larger surface-to-mass ratio (Øvrevik et al. 2015).

## Conclusions

To conclude, our findings indicate that particulate engine emissions from each type of biodiesel fuel induce cytotoxic effects in BEAS-2B and A549 cells, manifested either as cell death (apoptosis or necrosis), decreased protein concentrations, intracellular ROS production, as well as increased expression of cellular antioxidants and DNA damage-response genes. While many of the differences in effects between DEP from the different biodiesel blend percentage and biodiesel feedstock were statistically significant, the magnitude of these differences were rather marginal. Overall, this suggest that increasing the concentration of FAME in biodiesel from the current 7 to 20% or substituting FAME with HVO (below 20% blend) affects the toxicity from DEP emissions, but the biological significance of this may be moderate. However, as our results were obtained in in vitro systems based on immortalized cell lines, the findings should be interpreted with some caution. Other endpoints could potentially also be more affected by these alterations in biodiesel blends. As part of the FuelHealth project, in vitro genotoxicity and pro-inflammatory effects have been tested for the same DEP samples and in vivo effects of diesel exhaust from these biodiesel blends have been examined in a rat inhalation study (unpublished results). It is anticipated that the present toxicity results, in combination with the other studies performed as part of the FuelHealth project, will contribute to increased knowledge on the potential health impact of increasing the biodiesel concentration, replacing the current 1st-generation FAME biodiesel with 2nd-generation HVO biodiesel in commercial diesel fuels. A better understanding of the toxicity induced by DEPs from the combustion of various biodiesel fuels also will help to understand their contribution to the pathogenesis of disorders associated with particle exposure.

## Electronic supplementary material

Supplementary Table 1 (DOC 130 kb)

Supplementary Table 2 (DOC 128 kb)

Supplementary data 1 (GIF 257 kb)

High-resolution image (TIFF 284 kb)
