# Supplementary material for: A comparative analysis of in vitro toxicity of diesel exhaust particles from combustion of 1st- and 2nd-generation biodiesel fuels in relation to their physicochemical properties—the FuelHealth project
Source: Environ Sci Pollut Res Int. 2017 Jul 3;24(23):19357–74. doi: 10.1007/s11356-017-9561-9 (PMC5556143; doi:10.1007/s11356-017-9561-9)
Supplement: Supplementary file 1 — (DOC 130 kb) [file 11356_2017_9561_MOESM1_ESM.doc]

**Supplementary Table I.** Changes in gene expression in BEAS-2B cells after treatment with 50 µg/ml of three types of DEPs (B7-DEPs, B20-DEPs and SHB-DEPs) for 6 hours. Mean fold change values from three independent experiments are presented. Fold changes statistically significant in Student’s t-test are highlighted in green (up-regulated genes) or red (down-regulated genes).

| **Target Name** | **UniGene** | **B20-DEPs** | | **B7-DEPs** | | **SHB-DEPs** | |
| --- | --- | --- | --- | --- | --- | --- | --- |
|  |  | **Mean fold change** | **t-test**  **p-value** | **Mean fold change** | **t-test**  **p-value** | **Mean fold change** | **t-test**  **p-value** |
| ADM | Hs.441047 | 1 | 0,998 | 0,897 | 0,308 | 0,96 | 0,673 |
| AKR1B1 | Hs.521212 | 0,969 | 0,589 | 1,055 | 0,188 | 0,986 | 0,709 |
| AQP1 | Hs.76152 | 0,504 | 0,423 | 1,359 | 0,68 | 0,387 | 0,332 |
| AQP4 | Hs.315369 | 1,193 | 0,539 | 1,342 | 0,648 | 1,234 | 0,725 |
| ARNT | Hs.632446 | 0,845 | 0,193 | 0,949 | 0,707 | 0,888 | 0,153 |
| ATF4 | Hs.496487 | 0,964 | 0,097 | 1,133 | 0,077 | 1,01 | 0,771 |
| ATF6 | Hs.617868 | 1,015 | 0,825 | 1,034 | 0,625 | 0,997 | 0,962 |
| ATF6B | Hs.42853 | 0,942 | 0,324 | 0,876 | 0,141 | 0,909 | 0,171 |
| ATG12 | Hs.264482 | 0,977 | 0,454 | 1,029 | 0,504 | 0,968 | 0,243 |
| ATG5 | Hs.486063 | 1,013 | 0,642 | 0,944 | 0,642 | 1,017 | 0,634 |
| ATG7 | Hs.740389 | 1,008 | 0,837 | 0,959 | 0,671 | 0,998 | 0,976 |
| ATM | Hs.367437 | 0,907 | 0,611 | 1,023 | 0,913 | 0,948 | 0,725 |
| ATR | Hs.271791 | 0,935 | 0,392 | 0,92 | 0,298 | 0,879 | 0,138 |
| BBC3 | Hs.467020 | 0,858 | 0,353 | 0,663 | 0,255 | 0,846 | 0,103 |
| BECN1 | Hs.716464 | 0,977 | 0,543 | 0,98 | 0,656 | 0,967 | 0,411 |
| BID | Hs.591054 | 0,972 | 0,358 | 1,056 | 0,517 | 0,979 | 0,602 |
| BNIP3L | Hs.131226 | 0,963 | 0,105 | 0,941 | 0,36 | 0,932 | 0,034 |
| CA9 | Hs.63287 | 0,872 | 0,289 | 0,799 | 0,131 | 0,859 | 0,254 |
| CALR | Hs.515162 | 0,983 | 0,794 | 0,897 | 0,367 | 0,986 | 0,838 |
| CASP1 | Hs.2490 | 0,972 | 0,08 | 1 | 1 | 0,972 | 0,342 |
| CCL2 | Hs.303649 | 1,206 | 0,428 | 0,862 | 0,499 | 0,936 | 0,757 |
| CD40LG | Hs.592244 | 0,487 | 0,434 | 0,223 | 0,178 | 1,17 | 0,866 |
| CDKN1A | Hs.732576 | 0,989 | 0,86 | 1,034 | 0,703 | 0,93 | 0,32 |
| CHEK1 | Hs.595920 | 0,978 | 0,702 | 0,951 | 0,426 | 0,959 | 0,444 |
| CHEK2 | Hs.505297 | 0,984 | 0,772 | 0,946 | 0,441 | 0,95 | 0,391 |
| DDB2 | Hs.700338 | 0,939 | 0,22 | 0,913 | 0,139 | 0,889 | 0,07 |
| DDIT3 | Hs.505777 | 0,98 | 0,776 | 1,072 | 0,429 | 0,982 | 0,799 |
| DNAJC3 | Hs.59214 | 1,001 | 0,997 | 0,959 | 0,511 | 0,915 | 0,007 |
| EDN1 | Hs.713645 | 1,024 | 0,816 | 0,889 | 0,311 | 0,885 | 0,241 |
| EPO | Hs.2303 | 0,618 | 0,566 | 0,742 | 0,396 | 0,382 | 0,05 |
| FAS | Hs.244139 | 0,98 | 0,566 | 0,937 | 0,153 | 0,942 | 0,131 |
| FTH1 | Hs.712676 | 1,04 | 0,498 | 1,301 | 0,029 | 1,044 | 0,461 |
| GADD45A | Hs.80409 | 1,044 | 0,628 | 1,11 | 0,274 | 1,14 | 0,315 |
| GADD45G | Hs.9701 | 0,739 | 0,384 | 1,005 | 0,979 | 0,938 | 0,753 |
| GCLC | Hs.654465 | 1,311 | 0,091 | 1,842 | 0,014 | 1,542 | 0,022 |
| GCLM | Hs.315562 | 1,203 | 0,037 | 1,794 | 0,001 | 1,265 | 0,014 |
| GRB2 | Hs.444356 | 1,006 | 0,888 | 0,933 | 0,711 | 1,04 | 0,365 |
| GSR | Hs.271510 | 1,243 | 0,018 | 1,233 | 0,173 | 1,364 | 0,005 |
| GSTP1 | Hs.523836 | 1,021 | 0,655 | 1,101 | 0,442 | 1,029 | 0,486 |
| HMOX1 | Hs.517581 | 1,362 | 0,068 | 3,441 | 0,003 | 1,396 | 0,002 |
| HSP90AA1 | Hs.525600 | 0,951 | 0,31 | 1,032 | 0,464 | 1,029 | 0,493 |
| HSP90B1 | Hs.192374 | 0,966 | 0,377 | 0,972 | 0,279 | 0,916 | 0,011 |
| HSPA4 | Hs.90093 | 0,983 | 0,543 | 1,012 | 0,845 | 0,927 | 0,109 |
| HSPA4L | Hs.135554 | 1,032 | 0,382 | 1,003 | 0,963 | 1,055 | 0,138 |
| HSPA5 | Hs.743241 | 0,891 | 0,156 | 0,974 | 0,76 | 0,876 | 0,113 |
| HUS1 | Hs.152983 | 1,043 | 0,643 | 1,109 | 0,325 | 1,211 | 0,153 |
| IL1A | Hs.1722 | 0,967 | 0,506 | 1,072 | 0,268 | 1,063 | 0,283 |
| IL1B | Hs.126256 | 1,09 | 0,211 | 0,767 | 0,018 | 1,034 | 0,702 |
| IL6 | Hs.654458 | 0,988 | 0,889 | 1,016 | 0,864 | 0,982 | 0,837 |
| IL8 | Hs.624 | 1,163 | 0,189 | 1,11 | 0,356 | 1,017 | 0,836 |
| LDHA | Hs.2795 | 0,983 | 0,834 | 0,991 | 0,904 | 1,047 | 0,309 |
| MCL1 | Hs.632486 | 0,942 | 0,114 | 0,988 | 0,892 | 0,92 | 0,149 |
| MMP9 | Hs.297413 | 0,955 | 0,716 | 0,827 | 0,31 | 0,841 | 0,201 |
| MRE11A | Hs.192649 | 0,99 | 0,852 | 0,93 | 0,249 | 0,96 | 0,193 |
| NBN | Hs.492208 | 0,964 | 0,186 | 1,069 | 0,509 | 1,043 | 0,37 |
| NFAT5 | Hs.371987 | 0,906 | 0,264 | 0,982 | 0,872 | 0,956 | 0,563 |
| NQO1 | Hs.406515 | 1,095 | 0,019 | 1,375 | 0,002 | 1,096 | 0,019 |
| PARP1 | Hs.177766 | 0,977 | 0,753 | 1,021 | 0,786 | 1,044 | 0,503 |
| PRDX1 | Hs.731900 | 1,008 | 0,821 | 1,086 | 0,189 | 0,968 | 0,416 |
| PVR | Hs.171844 | 0,93 | 0,214 | 0,801 | 0,356 | 0,938 | 0,149 |
| RAD17 | Hs.16184 | 1,013 | 0,743 | 1,056 | 0,253 | 0,977 | 0,525 |
| RAD51 | Hs.631709 | 0,954 | 0,57 | 0,947 | 0,415 | 0,923 | 0,27 |
| RAD9A | Hs.655354 | 0,917 | 0,262 | 0,838 | 0,254 | 0,926 | 0,054 |
| RIPK1 | Hs.519842 | 0,981 | 0,773 | 1,013 | 0,851 | 1,004 | 0,938 |
| SERPINE1 | Hs.713079 | 0,952 | 0,361 | 0,75 | 0,111 | 0,836 | 0,005 |
| SLC2A1 | Hs.473721 | 0,896 | 0,13 | 0,723 | 0,217 | 0,929 | 0,221 |
| SLC5A3 | Hs.302742 | 0,894 | 0,187 | 0,791 | 0,047 | 0,911 | 0,243 |
| SQSTM1 | Hs.724025 | 1,021 | 0,655 | 1,116 | 0,043 | 0,987 | 0,721 |
| TLR4 | Hs.174312 | 0,924 | 0,222 | 0,808 | 0,028 | 0,882 | 0,106 |
| TNF | Hs.241570 | 1,398 | 0,074 | 1,247 | 0,331 | 1,186 | 0,4 |
| TNFRSF10A | Hs.213467 | 0,979 | 0,612 | 0,921 | 0,479 | 1,034 | 0,516 |
| TNFRSF10B | Hs.521456 | 0,942 | 0,304 | 0,88 | 0,18 | 0,946 | 0,255 |
| TNFRSF1A | Hs.279594 | 1,039 | 0,625 | 0,983 | 0,846 | 1,013 | 0,795 |
| TP53 | Hs.740601 | 0,966 | 0,399 | 0,955 | 0,714 | 0,986 | 0,482 |
| TXN | Hs.435136 | 0,978 | 0,268 | 1,092 | 0,249 | 0,976 | 0,275 |
| TXNL4B | Hs.134406 | 0,944 | 0,121 | 0,996 | 0,955 | 0,942 | 0,063 |
| TXNRD1 | Hs.654922 | 1,15 | 0,031 | 1,767 | 0,028 | 1,273 | 0,005 |
| ULK1 | Hs.47061 | 0,923 | 0,395 | 0,802 | 0,461 | 1,071 | 0,54 |
| VEGFA | Hs.73793 | 0,87 | 0,036 | 0,85 | 0,077 | 0,885 | 0,047 |
| XPC | Hs.739296 | 0,929 | 0,784 | 0,719 | 0,215 | 0,789 | 0,333 |
