# Supplementary material for: A comparative analysis of in vitro toxicity of diesel exhaust particles from combustion of 1st- and 2nd-generation biodiesel fuels in relation to their physicochemical properties—the FuelHealth project
Source: Environ Sci Pollut Res Int. 2017 Jul 3;24(23):19357–74. doi: 10.1007/s11356-017-9561-9 (PMC5556143; doi:10.1007/s11356-017-9561-9)
Supplement: Supplementary file 2 — (DOC 128 kb) [file 11356_2017_9561_MOESM2_ESM.doc]

**Supplementary Table II.** Changes in gene expression in A549 cells after treatment with 50 µg/ml of three types of DEPs (B7-DEPs, B20-DEPs and SHB-DEPs) for 6 hours. Mean fold change values from three independent experiments are presented. Fold changes statistically significant in Student’s t-test are highlighted in green (up-regulated genes) or red (down-regulated genes).

| **Target Name** | **UniGene** | **B20-DEPs** | | **B7-DEPs** | | **SHB-DEPs** | |
| --- | --- | --- | --- | --- | --- | --- | --- |
|  |  | **Mean fold change** | **t-test**  **p-value** | **Mean fold change** | **t-test**  **p-value** | **Mean fold change** | **t-test**  **p-value** |
| ADM | Hs.441047 | 1,038 | 0,403 | 1,102 | 0,053 | 1,107 | 0,105 |
| AKR1B1 | Hs.521212 | 0,977 | 0,357 | 1,001 | 0,989 | 1,024 | 0,327 |
| AQP1 | Hs.76152 | 0,963 | 0,847 | 0,868 | 0,702 | 0,86 | 0,455 |
| AQP4 | Hs.315369 | 1,428 | 0,203 | 1,168 | 0,295 | 0,989 | 0,968 |
| ARNT | Hs.632446 | 0,977 | 0,718 | 1,101 | 0,097 | 1,051 | 0,331 |
| ATF4 | Hs.496487 | 0,879 | 0,03 | 0,884 | 0,042 | 0,926 | 0,109 |
| ATF6 | Hs.617868 | 0,973 | 0,758 | 1,001 | 0,989 | 1,008 | 0,931 |
| ATF6B | Hs.42853 | 0,925 | 0,367 | 0,956 | 0,654 | 0,963 | 0,632 |
| ATG12 | Hs.264482 | 0,957 | 0,028 | 0,993 | 0,703 | 0,91 | 0,228 |
| ATG5 | Hs.486063 | 0,965 | 0,111 | 0,954 | 0,199 | 0,969 | 0,193 |
| ATG7 | Hs.740389 | 0,884 | 0,161 | 0,927 | 0,362 | 0,94 | 0,39 |
| ATM | Hs.367437 | 1,055 | 0,262 | 1,1 | 0,121 | 1,105 | 0,079 |
| ATR | Hs.271791 | 1,001 | 0,989 | 1,006 | 0,943 | 1,004 | 0,963 |
| BBC3 | Hs.467020 | 0,647 | 0,191 | 0,621 | 0,163 | 0,753 | 0,35 |
| BECN1 | Hs.716464 | 0,955 | 0,28 | 0,936 | 0,293 | 0,958 | 0,337 |
| BID | Hs.591054 | 1 | 0,999 | 1,011 | 0,755 | 0,986 | 0,651 |
| BNIP3L | Hs.131226 | 0,865 | 0,011 | 0,834 | 0,076 | 0,885 | 0,042 |
| CA9 | Hs.63287 | 0,867 | 0,093 | 0,736 | 0,031 | 0,907 | 0,222 |
| CALR | Hs.515162 | 0,993 | 0,925 | 0,982 | 0,85 | 1,019 | 0,828 |
| CASP1 | Hs.2490 | 1,081 | 0,747 | 1,091 | 0,682 | 1,034 | 0,876 |
| CCL2 | Hs.303649 | 1,444 | 0,017 | 1,616 | 0,031 | 1,437 | 0,045 |
| CD40LG | Hs.592244 | 1,73 | 0,425 | 0,612 | 0,487 | 0,946 | 0,954 |
| CDKN1A | Hs.732576 | 0,974 | 0,549 | 1,152 | 0,125 | 0,979 | 0,702 |
| CHEK1 | Hs.595920 | 1,02 | 0,088 | 1,053 | 0,047 | 0,997 | 0,795 |
| CHEK2 | Hs.505297 | 0,959 | 0,581 | 0,969 | 0,658 | 0,946 | 0,448 |
| DDB2 | Hs.700338 | 1,243 | 0,011 | 1,398 | 0,022 | 1,315 | 0,016 |
| DDIT3 | Hs.505777 | 0,742 | 0,1 | 0,686 | 0,209 | 0,849 | 0,219 |
| DNAJC3 | Hs.59214 | 0,916 | 0,027 | 0,958 | 0,493 | 0,933 | 0,051 |
| EDN1 | Hs.713645 | 0,985 | 0,667 | 0,989 | 0,756 | 1,004 | 0,937 |
| EPO | Hs.2303 | 0,919 | 0,769 | 1,023 | 0,956 | 1,573 | 0,224 |
| FAS | Hs.244139 | 0,983 | 0,749 | 1,047 | 0,482 | 0,948 | 0,381 |
| FTH1 | Hs.712676 | 0,999 | 0,969 | 1,036 | 0,349 | 1,008 | 0,429 |
| GADD45A | Hs.80409 | 1,069 | 0,723 | 1,108 | 0,566 | 1,101 | 0,66 |
| GADD45G | Hs.9701 | 1,182 | 0,526 | 1,565 | 0,164 | 1,146 | 0,595 |
| GCLC | Hs.654465 | 1,029 | 0,869 | 0,997 | 0,991 | 1,261 | 0,199 |
| GCLM | Hs.315562 | 1,065 | 0,195 | 1,107 | 0,14 | 1,066 | 0,192 |
| GRB2 | Hs.444356 | 0,909 | 0,194 | 0,928 | 0,231 | 0,985 | 0,734 |
| GSR | Hs.271510 | 0,983 | 0,928 | 0,991 | 0,944 | 1,091 | 0,5 |
| GSTP1 | Hs.523836 | 1,038 | 0,475 | 1,085 | 0,233 | 1,094 | 0,069 |
| HMOX1 | Hs.517581 | 1,204 | 0,008 | 1,221 | 0,015 | 1,378 | 0,023 |
| HSP90AA1 | Hs.525600 | 1,052 | 0,072 | 1,089 | 0,2 | 1,022 | 0,322 |
| HSP90B1 | Hs.192374 | 1,057 | 0,057 | 1,045 | 0,236 | 1,036 | 0,099 |
| HSPA4 | Hs.90093 | 1,041 | 0,382 | 1,045 | 0,296 | 1,025 | 0,577 |
| HSPA4L | Hs.135554 | 1,084 | 0,266 | 1,108 | 0,068 | 1,058 | 0,298 |
| HSPA5 | Hs.743241 | 0,996 | 0,914 | 1,056 | 0,186 | 1,044 | 0,141 |
| HUS1 | Hs.152983 | 1,078 | 0,792 | 1,11 | 0,636 | 1,303 | 0,253 |
| IL1A | Hs.1722 | 1,732 | 0,027 | 1,937 | 0,026 | 1,721 | 0,039 |
| IL1B | Hs.126256 | 1,12 | 0,68 | 1,194 | 0,538 | 1,002 | 0,994 |
| IL6 | Hs.654458 | 0,74 | 0,216 | 0,838 | 0,463 | 0,749 | 0,231 |
| IL8 | Hs.624 | 1,346 | 0,005 | 1,541 | 0,027 | 1,257 | 0,046 |
| LDHA | Hs.2795 | 1,014 | 0,65 | 0,978 | 0,548 | 1,053 | 0,252 |
| MCL1 | Hs.632486 | 0,903 | 0,132 | 0,905 | 0,143 | 0,875 | 0,091 |
| MMP9 | Hs.297413 | 1,039 | 0,621 | 1,047 | 0,507 | 0,97 | 0,62 |
| MRE11A | Hs.192649 | 1,044 | 0,224 | 1,008 | 0,834 | 1,002 | 0,953 |
| NBN | Hs.492208 | 0,936 | 0,261 | 0,994 | 0,927 | 0,913 | 0,107 |
| NFAT5 | Hs.371987 | 0,841 | 0,077 | 0,869 | 0,117 | 0,883 | 0,255 |
| NQO1 | Hs.406515 | 0,99 | 0,718 | 1,027 | 0,368 | 0,996 | 0,821 |
| PARP1 | Hs.177766 | 1,025 | 0,592 | 1,015 | 0,441 | 1,032 | 0,168 |
| PRDX1 | Hs.731900 | 1,046 | 0,427 | 1,041 | 0,387 | 1,023 | 0,708 |
| PVR | Hs.171844 | 0,819 | 0,06 | 0,844 | 0,155 | 0,865 | 0,107 |
| RAD17 | Hs.16184 | 1,042 | 0,379 | 1,009 | 0,904 | 0,979 | 0,724 |
| RAD51 | Hs.631709 | 1,017 | 0,656 | 0,991 | 0,845 | 0,985 | 0,691 |
| RAD9A | Hs.655354 | 0,799 | 0,115 | 0,768 | 0,077 | 0,884 | 0,297 |
| RIPK1 | Hs.519842 | 1,034 | 0,422 | 1,052 | 0,277 | 1,037 | 0,436 |
| SERPINE1 | Hs.713079 | 1,003 | 0,943 | 1,031 | 0,623 | 1,062 | 0,223 |
| SLC2A1 | Hs.473721 | 1,002 | 0,979 | 0,961 | 0,662 | 1,152 | 0,134 |
| SLC5A3 | Hs.302742 | 0,931 | 0,15 | 0,922 | 0,203 | 0,931 | 0,147 |
| SQSTM1 | Hs.724025 | 0,998 | 0,914 | 1,033 | 0,357 | 1,024 | 0,354 |
| TLR4 | Hs.174312 | 0,554 | 0,028 | 0,499 | 0,022 | 0,324 | 0,026 |
| TNF | Hs.241570 | 1,544 | 0,698 | 1,425 | 0,343 | 1,205 | 0,706 |
| TNFRSF10A | Hs.213467 | 0,871 | 0,336 | 0,925 | 0,136 | 0,94 | 0,297 |
| TNFRSF10B | Hs.521456 | 0,92 | 0,22 | 0,954 | 0,122 | 0,953 | 0,293 |
| TNFRSF1A | Hs.279594 | 0,909 | 0,339 | 0,888 | 0,261 | 0,938 | 0,5 |
| TP53 | Hs.740601 | 0,89 | 0,14 | 0,886 | 0,218 | 0,928 | 0,054 |
| TXN | Hs.435136 | 1,024 | 0,557 | 1,036 | 0,108 | 1,005 | 0,743 |
| TXNL4B | Hs.134406 | 0,892 | 0,105 | 0,908 | 0,148 | 0,882 | 0,088 |
| TXNRD1 | Hs.654922 | 0,957 | 0,404 | 0,967 | 0,546 | 1,037 | 0,389 |
| ULK1 | Hs.47061 | 0,744 | 0,142 | 0,703 | 0,078 | 0,87 | 0,366 |
| VEGFA | Hs.73793 | 0,951 | 0,38 | 0,972 | 0,686 | 1,038 | 0,491 |
| XPC | Hs.739296 | 1,122 | 0,596 | 0,889 | 0,516 | 0,939 | 0,753 |
