# Supplementary figures and images for: A comparative analysis of in vitro toxicity of diesel exhaust particles from combustion of 1st- and 2nd-generation biodiesel fuels in relation to their physicochemical properties—the FuelHealth project
Source: Environ Sci Pollut Res Int. 2017 Jul 3;24(23):19357–74. doi: 10.1007/s11356-017-9561-9 (PMC5556143; doi:10.1007/s11356-017-9561-9)

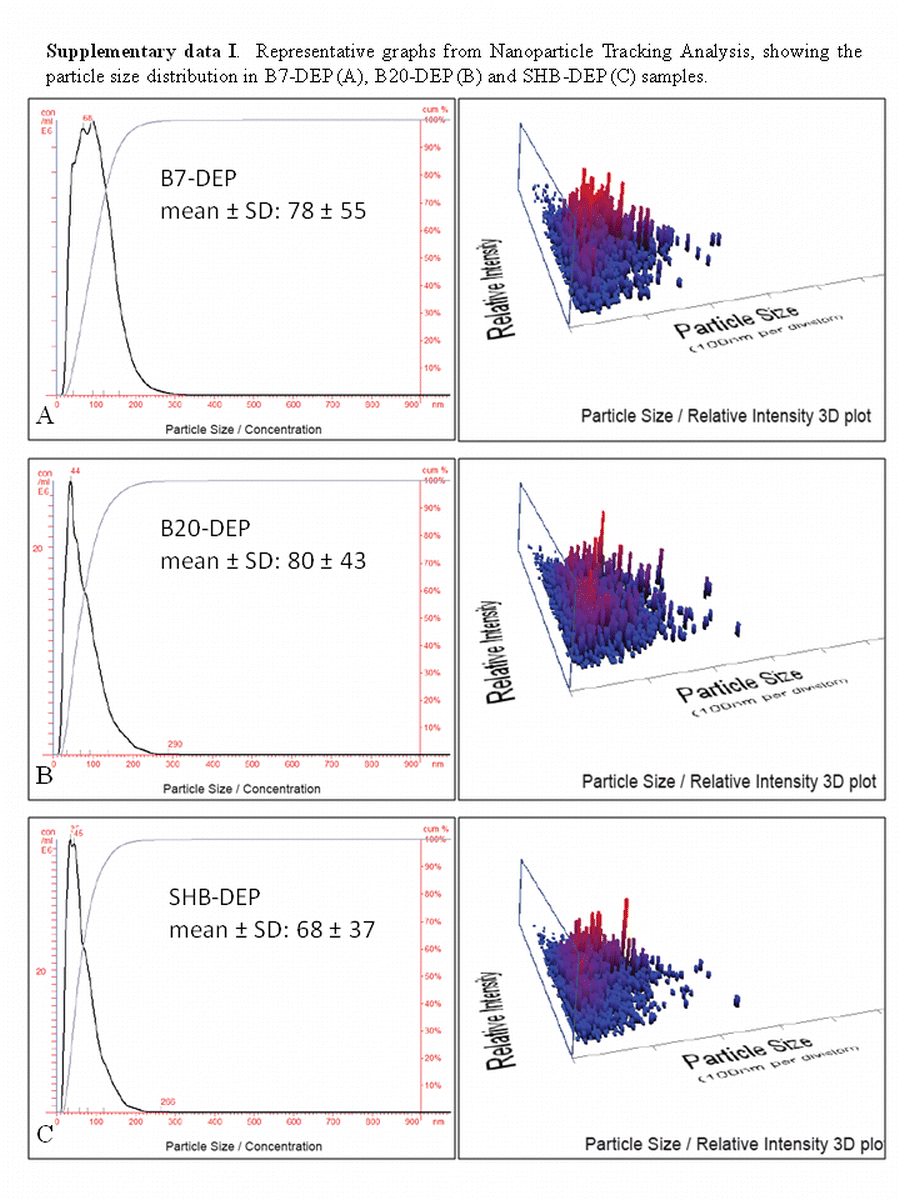

Supplement: Supplementary file 3 — (GIF 257 kb) [file 11356_2017_9561_Fig10_ESM.gif]

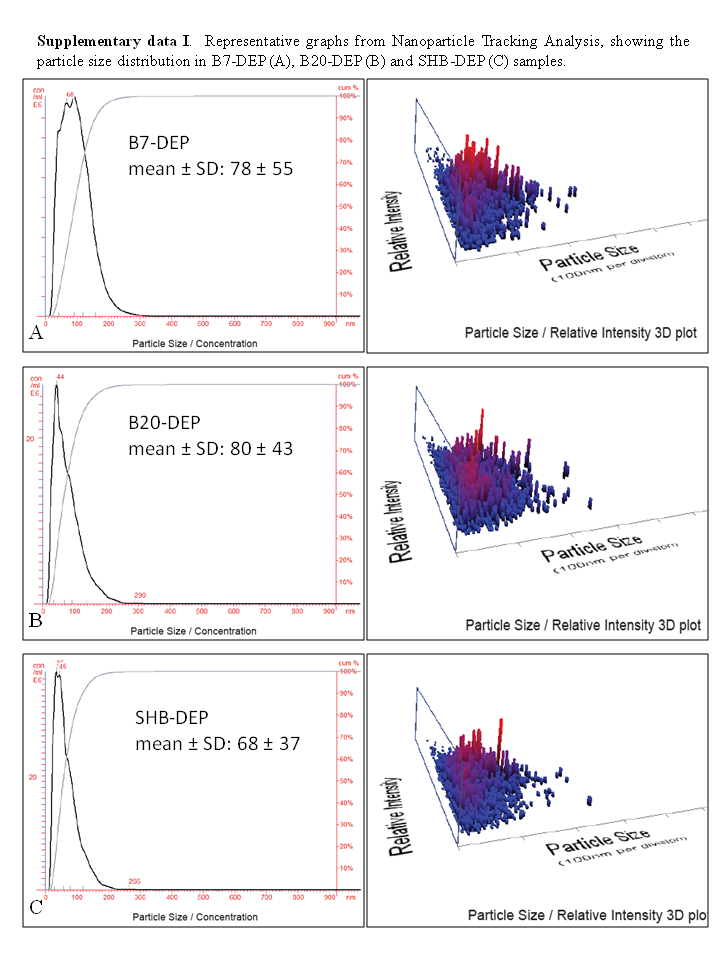

Supplement: Supplementary file 4 — High-resolution image (TIFF 284 kb) [file 11356_2017_9561_MOESM3_ESM.tif]
